# Electrospun Polymer Nanofibers with Antimicrobial Activity

**DOI:** 10.3390/polym14091661

**Published:** 2022-04-20

**Authors:** Irena Maliszewska, Tomasz Czapka

**Affiliations:** 1 Department of Organic and Medicinal Chemistry, Wrocław University of Science and Technology, 50-370 Wrocław, Poland; 2 Department of Electrical Engineering Fundamentals, Wrocław University of Science and Technology, 50-370 Wrocław, Poland

**Keywords:** electrospinning, nanofibers, antimicrobial activity, nanoparticles, bioactive agents

## Abstract

Nowadays, nanofibers with antimicrobial activity are of great importance due to the widespread antibiotic resistance of many pathogens. Electrospinning is a versatile method of producing ultrathin fibers with desired properties, and this technique can be optimized by controlling parameters such as solution/melt viscosity, feeding rate, and electric field. High viscosity and slow feeding rate cause blockage of the spinneret, while low viscosity and high feeding rate result in fiber discontinuities or droplet formation. The electric field must be properly set because high field strength shortens the solidification time of the fluid streams, while low field strength is unable to form the Taylor cone. Environmental conditions, temperature, and humidity also affect electrospinning. In recent years, significant advances have been made in the development of electrospinning methods and the engineering of electrospun nanofibers for various applications. This review discusses the current research on the use of electrospinning to fabricate composite polymer fibers with antimicrobial properties by incorporating well-defined antimicrobial nanoparticles (silver, titanium dioxide, zinc dioxide, copper oxide, etc.), encapsulating classical therapeutic agents (antibiotics), plant-based bioactive agents (crude extracts, essential oils), and pure compounds (antimicrobial peptides, photosensitizers) in polymer nanofibers with controlled release and anti-degradation protection. The analyzed works prove that the electrospinning process is an effective strategy for the formation of antimicrobial fibers for the biomedicine, pharmacy, and food industry.

## 1. Introduction

Nanofibers can be viewed as nanostructures and this category of nanomaterials includes nanotubes as well as nanorods. In addition, nanofibers can contain nanoparticles in their bulk or on their surface, forming so-called nanofibrous composite materials. Currently, well-known methods employed to fabricate polymeric nanofibers or nanostructures mainly include drawing, template synthesis, phase separation, self-assembly, and electrospinning [1,2,3,4,5].

(1) Drawing is the process that requires a viscoelastic material that can be severely deformed but is still cohesive sufficiently to withstand the stresses arising during pulling.
(2) Template synthesis involves using a template or mold to create the required material or structure.
(3) Phase separation is the method, the polymer is first mixed with a solvent and then subjected to gelation. the key mechanism of this process is to split the phases owing to physical incompatibilities. The solvent is then extracted, and the polymeric material is left.
(4) Self-assembly deals with the formation of nanofibers using smaller molecules as the basic building blocks. The intermolecular forces that occur in the process allow the smaller units to be brought together. The final shape of the fiber is strictly related to the shape of the constituent molecules.
(5) Electrospinning is the process of converting a liquid polymer solution into solid nanofibers by applying an electrical force in the presence of a strong electric field.

The different methods have certain advantages, but also some drawbacks, which are summarized in Table 1. Examples of the use of individual methods for the preparation of polymer nanofibers are given in Table 2. Among these methods, electrospinning is the only one that allows the process to be fully scaled. The most serious drawback of this process is the occurrence of instability of the jet from which the polymeric fiber is produced.

**Figure 1:**
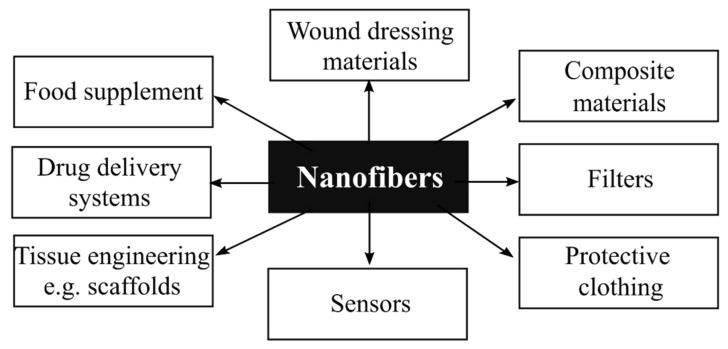
Examples of nanofiber applications in various areas of daily life (own elaboration).

Electrospinning enables the efficient fabrication of fibers with diameters ranging from a few nanometers to several micrometers. The electrospun nanofiber mats are characterized by high porosity, high gas permeability, and high surface area per unit mass. Due to the listed characteristics, nanofibers have found numerous applications in different areas of daily life. Some examples of nanofiber applications are shown in Figure 1. Apart from the applications presented in Figure 1, nanofibers, due to their unique physicochemical properties, are also utilized in flexible solid-state supercapacitors [12,13], sodium-ion and lithium-ion batteries [14], nanotube fabrication (e.g., polymer, metal, and metal oxide nanotubes) [15,16,17,18,19,20], nanogenerators for energy harvesting devices [21], environmental energy harvesting [22], electronics [23], wastewater treatment [24], and catalysis [25].

The numerous advantages of the electrospinning technique have led to the fabrication of nanofibers using a wide range of polymer solutions, i.e., synthetic and natural, as well as biodegradable and non-biodegradable [26,27,28,29]. Due to numerous environmental concerns, current research is focusing on the utilization of biodegradable polymers for the development of various biomedical applications and components for environmental protection [30,31,32,33,34,35,36,37,38]. Nowadays, biodegradable and biocompatible polymers, such as collagen, alginate, chitosan, cellulose acetate (CA), gelatin, poly(ethylene glycol) (PEG), poly(lactic acid) (PLA), poly(glycolic acid) (PGA), poly(D, L-lactide- co-glycolide) (PLGA), polycaprolactone (PCL), polybutylene succinate (PBS), polyvinyl alcohol (PVA), polyethylene oxide (PEO), and copolymers are widely employed to fabricate nanofibers [39,40,41,42,43,44,45,46].

## 2. Electrospinning as a Process for Nanofiber Fabrication

**Figure 2:**
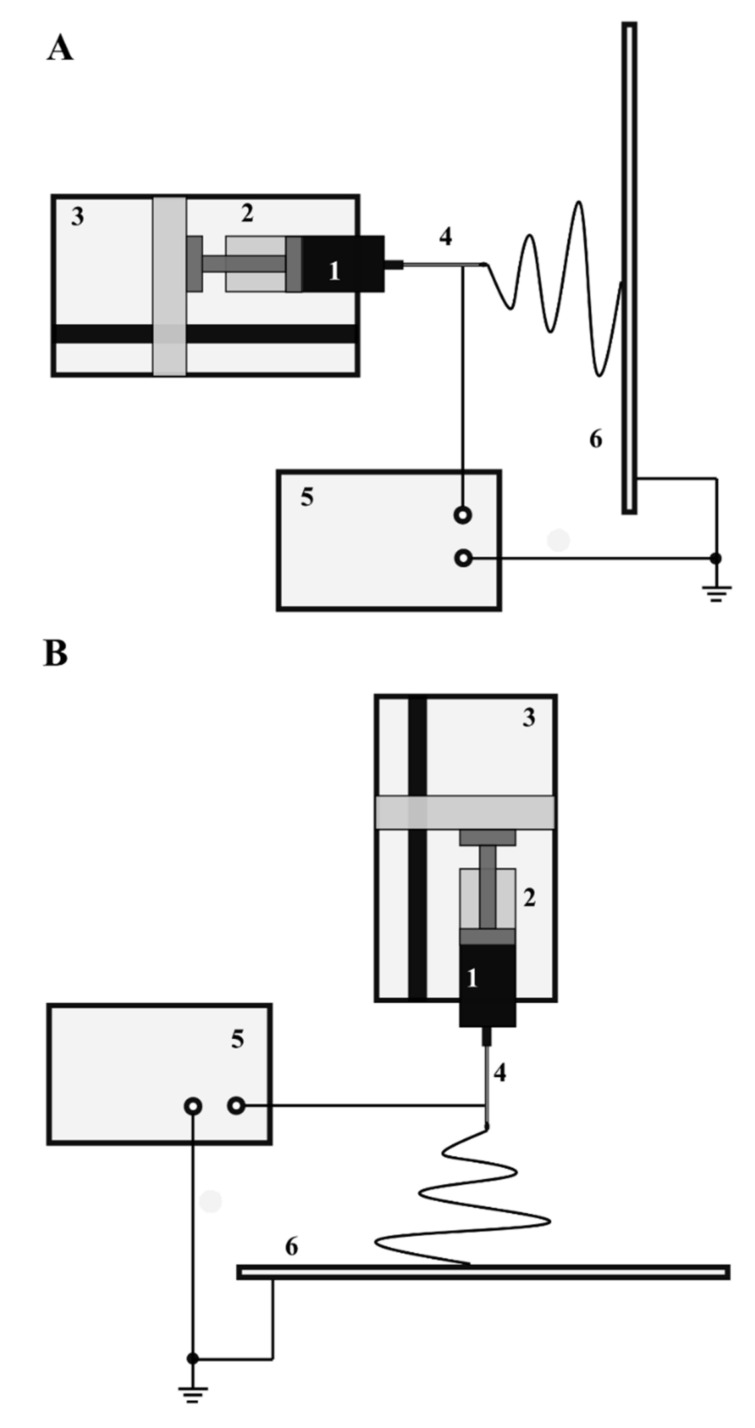
Horizontal (**A**) and vertical (**B**) arrangement for electrospinning process (1—polymer solution, 2—syringe. 3—syringe pump, 4—capillary (spinneret), 5—high voltage power supply, 6—collector) (own elaboration).

The schematic diagram of a typical electrospinning apparatus is shown in Figure 2. In this arrangement, the liquid polymer solution is placed in a syringe provided with a blunt-ended stainless steel capillary. The syringe is placed in a syringe pump that enables adjustment and precise control of the flow rate of the solution. The orientation of the syringe can be either horizontal (Figure 2A), or a vertical position is also used as presented in Figure 2B.

The polymer solution is pumped through a metal capillary, which is connected to a regulated high voltage power supply that provides voltages ranging from a few to tens of kilovolts depending on the experimental setup used. The process utilizes both positive and negative polarity voltages. The experimental system also includes a grounded collector as its final component. The presence of a capillary powered by a high voltage source located at a certain distance from the grounded collector allows a strong electric field to be generated between them and the fiber fabrication process to occur. The current values are usually very low and usually do not exceed a few microamperes. The electrospinning process is performed with different solutions of capillaries, also known as spinnerets. The most commonly used types of spinnerets are single jet, multi-jet, and co-axial [47]. Likewise, several types of collectors on which nanofibers are deposited can be identified. Exemplary collector arrangements are flat plates, (e.g., a single plate, with two or more parallel plates), meshes, rings, discs, and drums [47].

**Figure 3:**
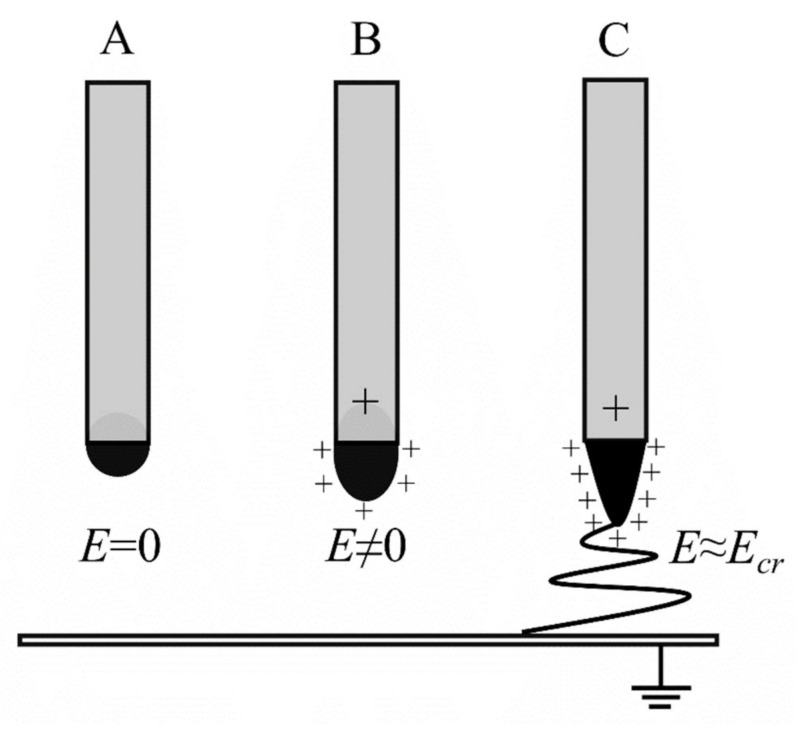
Schematic illustration of Taylor cone formation ((**A**)—a stable droplet of polymer solution with no electric field, (**B**)—a distorted droplet of polymer solution in the presence of an electric field, (**C**)—formation of nanofiber from Taylor cone for an electric field of critical value) (own elaboration).

A high voltage is applied to the polymer solution in the capillary and a strong electric field is created. The surface of the polymer at the end of the capillary forms a droplet due to the surface tension of the liquid as presented in Figure 3. In the absence of an electric field, the surface and the shape of the droplets are stable (Figure 3A). The application of an electric field causes the accumulation of electric charges on the surface of the polymer solution and the formation of significant electrostatic forces, (i.e., Coulomb forces), which are repulsive forces (Figure 3B). These forces lead to distortion, creating a conical droplet shape known as the Taylor cone. When the electric field exceeds a critical value, the surface forces of the solution are overcome and a charged stream of polymer solution is ejected from the tip of the Taylor cone (Figure 3C).

Due to the influence of an electric field, this jet moves towards a grounded collector. The charges present in the polymer jet are non-uniformly distributed, leading to rotational motions that change the shape of the jet. As a result of intense solvent evaporation, the polymer chains in the jet tend to stretch and orient themselves. The jet containing polymer particles is then deposited on the collector as a nanofiber of pure polymer. In the presence of a very strong electric field, depending on the properties of the solution, the jet may break up into small droplets, resulting in the deposition of polymer nanoparticles on the collector. This process is called electrospraying.

## 3. Factors Influencing the Electrospinning Process

It should be emphasized that the properties of the polymer solution, the parameters of the manufacturing process as well as the ambient conditions have a crucial impact on the morphology and diameter of the electrospun fibers. For this reason, the influence of the above-mentioned factors on the process is being discussed in detail. The solution parameters that play an important role in preparing the nanofibers are as follows. (1) Polymer concentration and solution viscosity. The concentration of the polymer is one of the most essential solution parameters because the concentration has the potential to significantly affect the viscosity of the solution and its surface tension. Most commonly, a low concentration results in the formation of beads on fibers (viewed as defects) in electrospinning. When the concentration increases, the viscosity increases correspondingly, and large diameter fibers are formed as a consequence [48]. (2) Surface tension. In electrospinning, electrostatic forces (repulsive Coulomb forces) must overcome the surface tension of the solution. Thus, if the surface tension is too high compared to the Coulombic forces, the jet formation mechanism may be blocked. The low surface tension of the solution allows the process to proceed at a lower capillary supply voltage. Surface tension has no clear effect on fiber morphology. Doshi et al. observed that reduction of surface tension results in avoidance of bead formation [49]. (3) Molecular weight. The molecular weight of polymers affects many important properties of the solution, such as dielectric strength, surface tension, viscosity, and electrical conductivity [50]. The low molecular weight of the polymer can lead to the formation of beads on the fibers and reduce the capability of obtaining nanofibers with a uniform diameter. On the other hand, the too high molecular weight can result in fibers characterized by high porosity and inhomogeneity of fiber morphology. (4) Solvent volatility. Solvent volatility performs a very important function in fiber formation. Highly volatile solvents generally facilitate the solidification of fibers, which can be positive in fabricating fibers with a homogeneous structure and very small diameter. (5) Electrical conductivity. The electrical charges, occurring when the capillary is powered by a high voltage source, significantly affect the mechanism of jet formation [40]. The high conductivity of the solution allows for an increased charge density in the solution, with the result that the size of the Taylor cone formed in the process changes, (i.e., is reduced). Consequently, the mentioned effect leads to a decrease in the diameter of the fibers [51]. It is observed that low conductivity solutions allow the formation of nanofibers without defects, while high conductivity solutions provide straight nanofibers [52]. 

The fabrication process parameters consist of the following. (1) Applied voltage/electric field strength. When the supply voltage of the capillary exceeds a critical value, the polymer solution changes its shape into a Taylor cone, from which the fiber evolves toward the collector. It can be concluded that an increase in the supply voltage, and thus the electric field strength, results in a decrease in the fiber diameter. (2) Flow rate. The flow rate represents the amount of solution being delivered to the capillary. When the flow rate is too high, the jet cannot be sufficiently stretched in a strong electric field, so there is an increase in the size of the fiber diameter [53]. (3) Capillary-to-collector distance. The distance between the capillary and the collector affects the morphology and diameter by determining the electric field strength in the experimental setup. In addition, this distance controls the solvent volatilization time. A small distance leads to an increase in the electric field strength and a decrease in the time for the jet to reach the collector. As a result, the diameter of the fibers may decrease when the mentioned distance is reduced. It should be emphasized that the distance between the capillary and the collector cannot be too small, as this will lead to a reduction in the process of solvent volatilization. As a consequence, undried fibers are deposited on the collector, which is an undesirable effect [49].

The ambient parameters comprise the following. (1) Humidity. Humidity has a direct effect on the morphology of the fibers. Higher humidity leads to an increase in the porosity of the fiber [54]. In addition, humidity influences the rate of solvent evaporation from the polymer solution, which in turn impacts fiber diameter and morphology. (2) Temperature. Temperature affects both the rate of solvent volatilization and also the viscosity of the solution [51] Increased temperature raises the rate of solvent volatilization and lowers the viscosity of the polymer solution, which significantly reduces the fiber diameter [54].

It is worth noting that residual solvents play an important role in polymeric materials used in medicine. Novel solvent-free electrospinning methods have emerged recently. However, the most widely applied method is electrospinning based on solution, which involves dissolving a polymer in a volatile organic solvent. As the process proceeds, the solvent evaporates and the fibers are deposited on the collection surface. It is important to consider the possibility of retaining the solvent in the fibers after fabrication because many solvents commonly used in electrospinning are toxic. Residual solvent in electrospun fibers may be a potential source of their cytotoxicity in medical applications, such as drug delivery systems or cell scaffolds. The investigation by Nam et al. was one of the first to examine solvent retention in electrospun fibers in detail [55]. D’Amato et al. studied the retention of both 1,1,1,3,3,3-hexafluoroisopropanol (HFIP) and chloroform in poly(lactic acid) (PLA) fibers and tested various methods to promote solvent removal [56]. Another study showed that HFIP retention affects the drug release kinetics of PLA fibers with the drug 6-aminonatecinomide (6AN) incorporated into the polymer matrix [57]. An alternative to toxic solvents is the use of water as a medium, and subsequent solidification of electrospun materials to improve their stability in physiological fluids. For this reason, natural polymers such as alginate, collagen, and cellulose, which are compatible with non-toxic solvents, are often used for biofabrication with living cells by non-toxic solvents, are often used for biofabrication using living cells via cell-electrospinning and bio-electrospraying methods [58]. In drug delivery systems, surface immobilization of the bioactive molecule after electrospinning is used. In this way, contact between the active molecule and the organic solvent can be avoided, preventing unwanted molecule degradation [59].

In recent years, it has been shown that electrospun nanometric fibers can be effective antimicrobial materials, as their physicochemical properties can be adapted to many applications requiring the necessary biocidal activity. Hamdan et al. [60] in an excellent review work considers the following properties of electrospun fibers to be key to achieving the expected antimicrobial activity: (1) fiber size (nanofibers in size from 100 to 1000 nm are similar to the size of bacteria, so they can strengthen the adhesion of bacteria); (2) surface area to volume ratio (smaller diameter nanofibers provide a higher surface to volume ratio for efficient encapsulation of antimicrobial agents); (3) porosity (high porosity allows greater loading of antimicrobials into the nanofibers, increases the surface area, which improves the attachment of bacteria on the surface of fibers); (4) interconnected pores (promote oxygen and nutrient exchange, enhance proliferation and provide structural stability, and enable the sustained release of antimicrobial agents).

## 4. Electrospun Fibers Based on Natural Antibacterial Polymers

**Figure 4:**
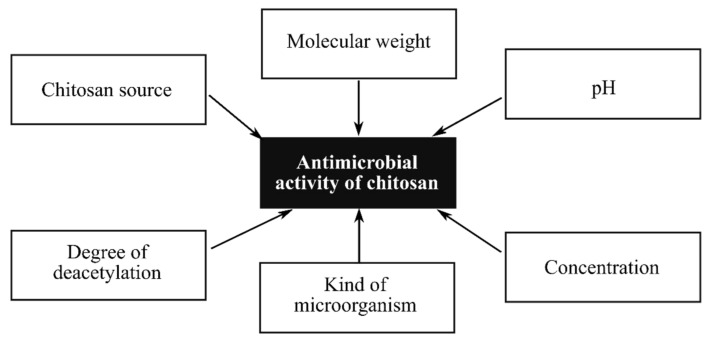
Selected factors influencing the antibacterial activity of chitosan (own elaboration).

Electrospun materials based on natural antibacterial polymers are intensively studied due to their biocompatibility, biodegradability, non-toxicity, non-mutagenicity, and non-immunogenicity. Chitosan, a partially N-deacetylated chitin derivative, is one of the best known natural polysaccharides, consisting of random mixtures of D-glucosamine and N-acetyl-D-glucosamine linked by a *β*- (1–4) bond in the polymer backbone and is the only one charged positively natural alkaline polysaccharide described [61]. This polymer contains three types of reactive functional groups, an amino group at the C-6 position and both primary and secondary hydroxyl groups at the C-6 and C-3 positions, respectively. Chitosan is considered a weak base and is insoluble in water and organic solvents. However, it is soluble in dilute aqueous acidic solutions (pH < 6.5), which can convert glucosamine units into the soluble form of R-NH_3_ [62]. It is known that this polymer exhibits biocidal properties only in an acidic medium, which is usually related to the poor solubility of chitosan at high pH. Chitosan inhibits the growth of many bacteria, fungi, and yeasts, although the activity of chitosan against Eucaryotic microorganisms is less effective compared to its activity against bacteria [63]. It is considered that the antimicrobial activity of chitosan is persuaded by its cationic behavior and is attributed to the polycationic nature of this polymer. Several mechanisms for the antimicrobial activity of chitosan have been proposed, including [64]: (1) interaction of positively charged chitosan molecules with negatively charged groups on the surface of the bacteria, which results in a disturbance of the permeability of the cell membrane; (2) interaction of diffused hydrolysis products with DNA, which leads to inhibition of mRNA and protein synthesis; (3) chelation of nutrients and essential metals; (4) creating a “chitosan membrane” on the cell surface that prevents nutrients from entering the cell or acts as an oxygen barrier that can inhibit the growth of aerobic bacteria. As mentioned above, chitosan is soluble in organic acids such as aqueous solutions of formic, acetic, and lactic acids. By including a limited amount of acid, chitosan is soluble in water-ethanol-methanol and -acetone mixtures. Free amino groups make chitosan a positively charged polyelectrolyte with a pH of 2 to 6, which results in chitosan solutions being highly viscous [44]. In addition, strong hydrogen bonds form a three-dimensional network and prevent the movement of polymer chains under the influence of an electric field [65]. Chitosan electrospinning is generally considered to be a complicated technique. Recently, Antanaby et al. [66] reviewed chitosan electrospinning for antimicrobial applications and showed the current trends in this field. In addition, the parameters influencing the antibacterial properties of chitosan and the problems associated with the electrospinning process of this polymer were discussed. Two main aspects of the use of chitosan for electrospinning nanofibers were indicated. Firstly, attention should be paid to the factors influencing the antimicrobial activity of this polymer (Figure 4). Moreover, it is significant that the molecular weight of the polymer reflects the number of entanglements of the polymer chains in the solution and plays a significant role in the electrospun into nanofibers. High polymer molecular weight produces large diameter fibers, while electrospinning of a solution with a polymer of too low molecular weight gives spheres instead of fibers [67]. Second, it should be taken into account that the original chitosan-based nanofibers without further processing exhibit some disadvantages, including poor wet stability and low mechanical properties in aqueous solution, negatively affecting their use as engineering antimicrobial materials. This problem can be solved by mixing chitosan with polymers such as polyethylene oxide (PEO), polyvinyl alcohol (PVA), polylactide (PLA), and polycaprolactone (PCL) to obtain composite nanofibers with the desired bactericidal properties [68]. Chitosan can also be successfully blended with other natural biopolymers, such as collagen, which are more easily electrospun [43]. This problem can be also solved by applying coaxial electrospinning. In this technique, chitosan and another polymer solution are spun through a spinning die composed of two coaxial capillaries to obtain chitosan-based nanofibers with a core-shell structure [69,70]. Crosslinking is an important method for improving the wet resistance and stability of electrospun chitosan-based nanofibers. Various cross-linking techniques and cross-linking agents for producing chitosan nanofibers have been described but chemical cross-linking is the most common method of modification of chitosan nanofiber membranes [71,72,73]. Chemical cross-linkers are effective in linking molecules to increase wet stability and stability of mechanical properties, but some authors indicated that this method may lead to increased cytotoxicity due to the potential toxicity of chemical cross-linkers [71]. On the other hand, Zhou et al. studied chitosan/PVA nanofibers, which were cross-linked using an aqueous glutaraldehyde solution and it was shown that these crosslinked nanofibers were nontoxic to L929 cells as well as exert good in vitro biocompatibility [74].

A careful analysis of the available literature data showed that the most common anti-microbial nanofibers based on chitosan contained not only natural/synthetic polymers, but also antibacterial agents, including metal/metal oxide nanoparticles, antibiotics, or biologically active particles. Abdelgawad et al. [75] described the possibility of obtaining nanofibrous chitosan/polyvinyl alcohol (PVOH) fibers with the addition of AgNPs. The antibacterial test showed that these fibers were characterized by sufficient resistance to *E. coli* as well as bacterial mortality was found to be reduced with increasing chitosan content (above 20%) in the system. Several years ago, there were reports of advanced biomedical devices consisting of two layers, the first of electrospun chitosan/TiO_2_ fibers, and the second of the extracellular matrix derived from adipose tissue [76]. These interesting materials showed a low percentage of bacterial penetration (*E. coli* and *S. aureus*) due to the synergistic effect of chitosan and TiO_2_. Additionally, in vivo analysis combined with appropriate histological and immunofluorescence staining proved the ability of the obtained mats to heal wounds within just 21 days [77]. It has been proven that the addition of ZnO nanoparticles to chitosan-based fibers significantly enhances the antimicrobial effect of these materials [78]. It was observed that the chitosan fibers exhibited a minimal inhibitory concentration (MIC) value of 130 and 190 μm/mL for *E. coli* and *C. albicans*, respectively. The composite fibers showed lower MIC values (110 and 160 µm/mL) for *E. coli* and *C. albicans*, respectively [78]. Chitosan has also been proposed as a matrix for electrospinning of Garcinia mangostana (GM) extracts [79]. This extract was loaded onto an electrospun mat of chitosan-ethylenediaminetetraacetic acid/polyvinyl alcohol (CS-EDTA/PVA) and the bactericidal effect against *S. aureus* and *E. coli* was described [79].

Zupančič et al. [80] reported novel metronidazole-loaded chitosan/PEO nanofibers for the treatment of local wound infection. A drug release study of 15% metronidazole loaded chitosan/PEO nanofiber shows a burst release of 60% in 10 min followed by 95% in the next 2 h. Sadri et al. [81] also electrospun the CS/PEO nanofibers with cefazolin drug to study its microbial property and found that 1% cefazolin loaded CS/PEO showed the antimicrobial activity against the *S. aureus* and *E. coli*.

Biologically active green tea extract was added to the electrospinning chitosan/PEO nanofiber by Sadri et al. [82]. The nanofiber mat prepared in this protocol was treated with glutaraldehyde vapors to improve its hydrophilicity property. The obtained nanofiber showed antibacterial activity against Gram-positive and Gram-negative bacteria [82].

Other naturally occurring or biological polymers, such as cellulose derivatives and poly (L-lysine) have also been studied as antimicrobial materials, but only the latter showed significant antimicrobial activity and good biocompatibility [83,84].

Biocidal materials were also obtained using electrospun cellulose derivatives (the most commonly used is cellulose acetate; cellulose is difficult to spin without the use of strong and harmful solvents). Cellulose acetate (CA) is a cheap cellulose derivative and shows good spinnability using a variety of solvents, (e.g., N, N-dimethylformamide, acetone, acetic acid, and mixtures thereof). In the form of fibers, CA nanofibers are characterized by a high surface-to-volume ratio, flexibility, high porosity, exceptional stiffness, and tensile strength compared to other known forms of CA [84]. However, to obtain antimicrobial properties, it is necessary to incorporate biocidal agents such as metallic nanoparticles [85,86,87,88] or photosensitizers [89].

## 5. Electrospun Fibers Based on Synthetic Polymers

It seems that electro-spun fibers based on antibacterial synthetic polymers have been more intensively studied due to a wide range of raw materials for their production, and the versatility in terms of chemical modifications. 

The production of antimicrobial nanofibers generally follows the strategy of incorporating biocide into the fibers. This can be achieved by uniformly mixing the active agent in the polymer solution before electrospinning, entrapping the active agent in the fiber core by coaxial electrospinning, entrapping the active agent in nanostructures before dispersing it in the electrospinning solution, or attaching the active agent to the surface of the fiber. Until now, various active substances including antibiotics, biocides, metallic nanoparticles, metal oxide nanoparticles, and natural bioactive compounds have been used.

In the case of “enrichment” of polymers with antibiotics, the drug was most often dissolved in the same or a different solvent as the polymer and slowly added to the polymer solution with stirring to form a homogeneous solution before electrospinning. In the past decade, several hydrophilic and hydrophobic antibiotics (tetracycline, cefoxitin, mupirocin, ciprofloxacin, gentamycin, ampicillin, etc.) have been introduced into various polymer nanofibers (PLA, PLA/PCL, PLGA, coPLA, etc.), using this simple protocol [90,91]. This method can accommodate a large range of the number of antibiotics incorporated into the nanofibers by adjusting the initial drug concentration in the electrospinning solution. The inclusion of antibiotics in the polymer solution may have some effect on the electrospinning of the polymer and the morphology of the nanofibers due to changes in the viscosity, surface tension, and conductivity of the solution. For example, Kim et al. [92] showed that the addition of sodium cefoxitin increased the conductivity and electrospinability of the PLGA/PLA/PEG-b-PLA solution, allowing the production of more uniform nanofibers, and decreased the fiber diameter in a concentration-dependent manner [92]. Mixing antibiotics in a polymer solution before electrospinning is a simple method of loading large amounts of drugs into any polymer nanofibers, but this protocol has a serious drawback. It has been shown many times that nanofiber antibiotics tend to leach quickly in an aqueous solution, and this phenomenon has been called burst release [93]. Various strategies have been used to provide a more sustained release. One popular method is the use of coaxial electrospinning technology in which the outer solution contains a polymer and the inner solution contains an antibiotic. Another approach to achieve sustained release is to adsorb or encapsulate the drug into a nanostructure before dispersing it in the polymer solution.

Many biocides, such as QAC, triclosan, chlorhexidine, and PHMB, exhibit a broad spectrum of antimicrobial properties against Gram-positive and Gram-negative bacteria. Taking these properties into account, it turned out that they can also be incorporated into nanofibers [94]. As with antibiotics, these low molecular weight structures have typically been suspended in polymer solutions before electrospinning. These cationic substances significantly increased the conductivity of the electrospinning solutions and caused up to a 20% reduction in the diameter of the fibers, but did not significantly affect the crystallinity of the obtained fibers [94]. It should be noted that such mixing almost always results in a rapid release of the active agent from the nanofibers in aqueous solutions. However, some of these biocides (e.g., chlorhexidine) have functional groups in their structures that can be used to attach to the fiber surface to slow down the release process [95].

The International Health Organization has indicated that in all parts of the world, antibiotic resistance is reaching dangerously high levels and that new mechanisms of microbial resistance to antibiotics are threatening the successful treatment of many infectious diseases. Without urgent action, it is believed that we are moving towards a post-antibiotic era where widespread infections and minor injuries can kill again. In response to the expanding presence of ESCAPE pathogens (this is an acronym for six highly pathogenic and antibiotic-resistant bacteria (*Enterococcus faecium*, *Staphylococcus aureus*, *Klebsiella pneumoniae*, *Acinetobacter baumannii*, *Pseudomonas aeruginosa*, and *Enterobacter spp.*); it has been recognized that this group of bacteria may “escape” from commonly used antibiotics due to their increasing multidrug resistance), contemporary research trends in the fabrication of electrospun nanofibers exhibiting antimicrobial activity have focused on metal/metal oxide nanoparticles and natural biocides (especially of plant origin).

## 6. Antimicrobial Effect of Electrospun Nanofibers Loaded with Metallic Nanoparticles

Many electrospun antimicrobial fibers are based on metal or metal oxide nanoparticles (e.g., silver, copper, ZnO, TiO_2_) that have been used for centuries on a macroscale. These nanofibers are an example of new delivery systems based on the reservoir concept, in which the polymer structure surrounds the reservoir with a release rate modulated by the rate of polymer degradation, the rate of diffusion, or detachment of the surface coating [96]. There are two techniques to incorporate nanoparticles for electrospun nanofibers, namely the direct and the indirect method. The direct method is the most commonly used and involves the direct dispersion of nanoparticles in the electrospinning polymer solution (homogeneous dispersion is prepared). The properties of the obtained fibers can be easily modified by changing the number of nanoparticles added. When one solvent is not suitable for dissolving the polymer and nanoparticle dispersion, two miscible solvents are most often used to individually dissolve/disperse the nanoparticles, and then they can be mixed. There are also known procedures for adding surfactants to modify the surface of nanoparticles to facilitate their dispersion [97]. It has been found that some nanoparticles may be present on the surface of the fiber and some may be concentrically arranged (the position depends on the fiber diameter and nanoparticles and treatment conditions). If the diameter of the introduced nanoparticle is below the diameter of the nanofibers, it is known that the nanostructures are arranged randomly. When a mixture of two or more solvents is used, positioning nanoparticles depends on the vapor pressure. Several metal/metal oxide nanoparticles have been electrospun in different diameters and with different types of polymers using this direct method of nanoparticle incorporation. It has been well documented that for some nanoparticles (not only metallic nanoparticles but also drug nanoparticles) that are not uniformly dispersed in the electrospinning solution, the surface treatment becomes a necessary step for incorporation into nanofiber mats [97]. Based on different methods for surface treatment nanoparticles can be formed on the surface or within the nanofiber. Surface treatment of electrospun nanofibers is an easy method to adhere nanoparticles to a surface. Generally, in the case of nanoparticle surface loading, the nanofibers are immersed in colloidal nanoparticle dispersions to allow the surface adsorption of these nanoparticles by various interactions. Electrostatic forces, hydrogen bonds, and interactions between different functional groups are just some of the interactions responsible for this type of adsorption. Mahanta et al. [98] demonstrated the increased ability of electrospun PVA nanofibers to adsorb gold and silver nanoparticles after modification of surface hydroxyl groups with thiol and amine groups. In situ reduction, hydrothermal assisted process, ultrasound, and sputtering etching are some of the indirect methods used for surface fabrication of nanoparticles to nanofibers [99,100,101]. Another way of incorporating nanoparticles into nanomaterials is the formation of nanoparticles in electrospun nanofibers. In this technique, metal precursors are first electrospun to form nanofibers, and then other treatments (laser ablation, gas–solid reaction, etc.), are performed to form nanoparticles within nanofibers [97].

### 6.1. Silver Nanoparticles

Silver nanoparticles (AgNPs) are the best known antimicrobial nanostructures characterized by a wide spectrum of antimicrobial activity [102] and a rare incidence of resistance [103]. Due to the unique antimicrobial bioactivity of silver nanoparticles, these structures can be widely used in many applications, becoming especially important in the biomedical industry [104]. Despite many studies, the mechanism of silver’s antimicrobial activity is still under discussion. Currently, there are several possible mechanisms of destroying pathogenic cells [103]: (1) silver ions interact with the thiol groups (–SH) of proteins (enzymes) that are essential for the bacterial respiration and transport of important substances across the cell membrane and within the cells; (2) silver ions also penetrate bacterial cells and interact with compounds containing phosphorus groups (DNA, proteins) and can inhibit replication process and respiratory function of the cell; (3) silver ions become bound to the bacterial cell wall and outer bacterial cell, thus altering the function of the bacterial membrane; (4) silver induces the release of reactive oxygen species (ROS) characterized by a strong bactericidal effect, and (5) silver disrupts cell signaling [105]. One of the strategies for the production of nanofibers containing silver nanoparticles relies on the direct incorporation of silver nitrate (AgNO_3_) by mixing with a polymer solution, followed by ultraviolet photoreduction [106,107,108], thermal reduction [109], or the well-known silver mirror reaction [110]. As expected, the antimicrobial activity of electrospun scaffolds was related to the concentration of AgNO_3_. For example, Liang et al. [111] investigated poly (etheramide) fibers loaded with AgNPs using 0.15% AgNO_3_ in a polymer solution and showed that the obtained material inhibited the growth of *S. aureus* and *E. coli* at the level of 99.99%. At the concentration of 0.05%, the bactericidal activity of these fibers significantly decreased (over 20%) [111]. For the UV irradiation nanoparticle preparation method, the polymer solutions were mixed with AgNO_3_ before the electrospinning process [106,107]. However, Phan et al. [108] applied the method of AgNO_3_ incorporation into nanofibers after the electrospinning process. The synthesized silver/polyacrylonitrile nanocomposite membranes showed excellent antibacterial activity against *E. coli* and *Bacillus subtilis* [108]. The silver mirror reaction (SMR) method was used for the modification of electrospun polyacrylonitrile (PAN) nanofibers by Shi et al. [110]. The obtained results indicated that PAN nanofibers loaded with silver nanoparticles exhibited excellent antimicrobial activity against *E. coli*, *S. aureus* and the fungus *Monilia albicans*. Jatoi et al. [109] presented study on generation of silver nanoparticles (AgNPs) on cellulose nanofibers by thermal treatment and DMF as reducing agents. The cellulose nanofibers were prepared by deacetylation of electrospun cellulose acetate (CA) nanofibers which were subsequently coated with silver using AgNO_3_ followed by thermal and DMF induced reduction processes. The bactericidal activity of these fibers was confirmed against *S. aureus* and *E. coli*. Noteworthy is also the method described by Du et al. [112]. In this study polyvinyl alcohol (PVA) nanofiber membranes containing silver nanoparticles were produced by a combination of electrospinning and a green reduction approach (polyphenols isolated from green tea were used to reduce silver ions). It was shown that the mass ratio of the polyphenols to AgNO_3_ plays a key role in controlling the size of the obtained silver nanoparticles. Interestingly, multilayer fabrics with a layer of a nanofiber PVA/AgNPs membrane applied to cotton as a substrate have been developed and applied to the shoe insoles. Manufactured shoe insoles with functional PVA nanofibers showed remarkable antibacterial activity against *S. aureus* and *E. coli*, (i.e., the mortality rate was above 99%).

On the other hand, the literature describes strategies for combining electrospun nanofibers and AgNPs that cover the surface of nanofibers or are embedded in a mass of the polymer. A simple method of producing nanofibers containing silver nanoparticles is the direct mixing of fabricated AgNPs in a polymer solution before electrospinning [113]. In this strategy, silver colloidal solutions are most often preferred for easy incorporation into nanofibers and various electrospun polymer systems with AgNPs such as polyvinylidene fluoride (PVDF) [114], PVA/poly(urethane) (PU) [115], nylon [116,117] and poly(vinylpyrrolidone) (PVP) [118], poly(vinyl) alcohol (PVA) [119] that have already been described. Table 3 lists the additional examples of electrospun silver-containing nanofibers with antimicrobial properties.

**Figure 5:**
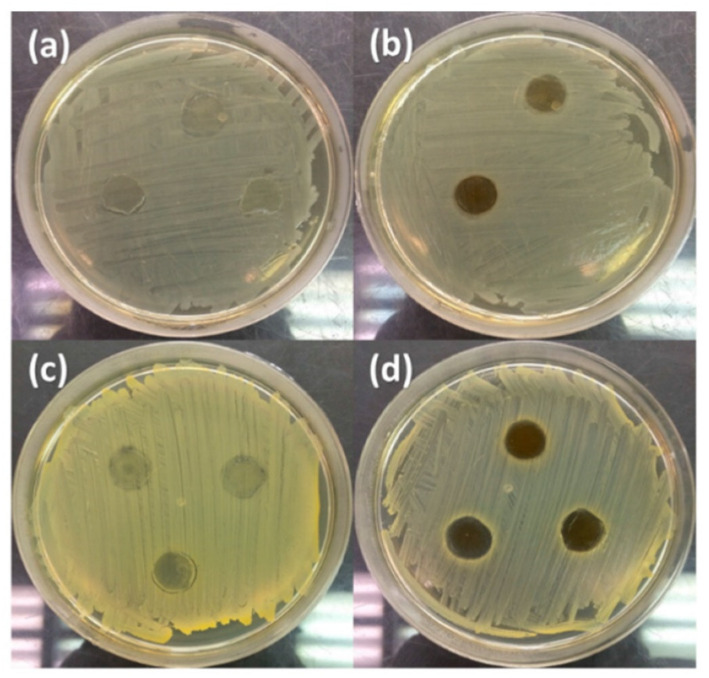
Photographs of the inhibition zones of PVP/CNC against *E. coli* (**a**) and *S. aureus* (**c**); and PVP/CNC-4%/AgNO_3_-0.34% against *E. coli* (**b**), and *S. aureus* (**d**). Reproduced from Ref. [119].

Studies of antimicrobial activity have usually been carried out by diffusion techniques and it has been shown that the nanofibers loaded with silver nanoparticles exhibited excellent antimicrobial properties. As can be seen in Figure 5, significant zones of growth inhibition of both Gram-positive and Gram-negative bacteria were observed [119].

It was pointed out that the loaded amount of AgNPs can vary significantly from 0.1 to 30 wt.% total weight of the polymer, which resulted in the formation of fibers of various diameters. It was observed that the higher concentration of AgNPs in the polymer solution resulted in obtaining higher conductivity and promoting smaller diameters of the fibers [114,128]. For example, Lopez-Esparza et al. [129] studied the antimicrobial silver nanoparticles embedded in PCL nanofiber mat for strong resistance against Gram-positive and Gram-negative bacteria. It was shown that the increased concentration of Ag (1–100 mM) helped to obtain nanofibers of smaller diameter (159 ± 79 nm), whereas the lower concentration of AgNPs resulted in the formation of thicker PCL nanofibers (234 ± 66 nm). These fibers showed sufficient resistance to pathogenic bacteria, as such *S. aureus*, *E. coli*, *P. aeruginosa*, *S. pyogenes*, and *K. pneumonia*. The interesting poly (ε-caprolactone) (PCL) microfibers without Ag on the surface were described by Bhullar et al. [130]. The *in-vitro* diffusion study exhibited controlled release of silver nanoparticles from the hybrid constructs and demonstrated high antibacterial activity against *S. aureus* and *E. coli*. Rzayev et al. [120] described the production of poly(vinyl alcohol-co-vinyl acetate)/octadecylamine-montmorillonite) fibers loaded with AgNPs, which showed high activity against yeasts of the genus *Candida* (*C. albicans*, *C. tropicalis*, *C. glabrata*, *C. keyfr*, and *C. krusei*), and bacteria (*S. aureus* and *E. coli*). The authors proved that the release of Ag(I) ions give the bactericidal effect of the obtained fibers. Moreover, the internalization of AgNPs into the fibers allowed for a longer bioavailability of silver on the application side, as the release of ions depended on the degradation time of the fibers. Lala et al. [131] presented a comparative study of the antimicrobial activity of three different nanofibers, i.e., CA, PAN, and PVC, with various amounts of AgNO_3_ treated with UV-irradiation. Electrospun nanofibers were tested against two different strains of Gram-negative bacteria, i.e., *E. coli* and *P. aeruginosa*.

Castro-Mayorga et al. [132] presented electrohydrodynamic processing, which combines the electrospraying and electrospinning techniques to produce a multilayer system comprising a poly(hydroxy alkanoate) (PHA) substrate and an electrospun PHA coating containing AgNPs. These materials reduced the *S. enterica* planktonic population below the detection limits at a very low silver concentration of 0.002% wt. It is also worth mentioning that AgNO_3_ is not always the best precursor for obtaining nanometric silver. Dolina et al. [133] prepared electrospun polyurethane nanofibers doped with silver nanoparticles derived from three different silver precursors, as well as silver nanoparticles with a zero valence. It turned out that the best results from all tested combinations were obtained with silver behenate-doped nanofibers. These fibers had a homogeneous coating of nanoparticles and were characterized by bactericidal activity against *E. coli*.

**Figure 6:**
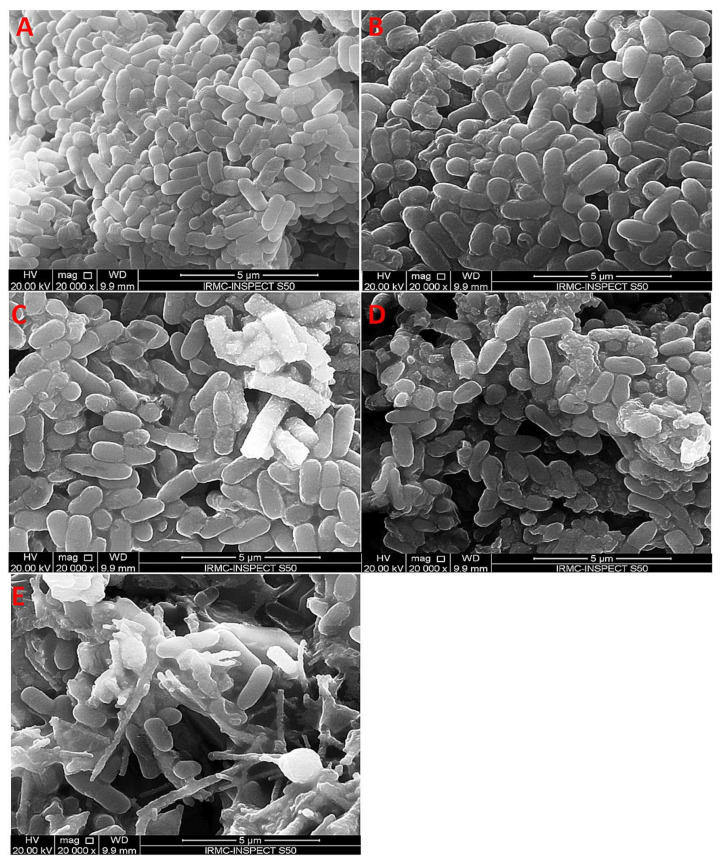
Effect of electrospun TiO_2_ NPs on the morphological aspects of *P. aeruginosa* as examined by scanning electron microscopy: (**A**) control without any treatment and treated with TiO_2_ calcined in (**B**) 100% Air, (**C**) 50% Air, and 50% Argon, (**D**) 25% Air and 75% Argon; and (**E**) 100% Argon. Reproduced from Ref. [135].

In recent years, it has also been shown that electrospun nanofibers can be doped not only with silver nanoparticles but also with other metallic particles (or metallic oxides). Nthunya et al. [134] reported on the green synthesis of low-swelling uniformly-sized chitosan (CTS)-based nanofibers decorated with silver (Ag) and silver/iron (Ag/Fe) nanoparticles [134]. These fibers were achieved by electrospinning a solution of CTS blended with varying amounts of polyacrylamide (PAA), polyethylene glycol (PEG), and Ag(I) or Ag(I)/Fe(III) ions. To obtain metallic nanoparticles, the nanofibers were subjected to UV irradiation in ionized water vapor at a low temperature. The biocidal effect of the Ag and Ag/Fe NPs supported on the CTS-based nanofibers was studied using Gram-positive bacteria: *B. cereus*, and *E. faecalis*, as well as Gram-negative rods: *E. coli*, *K. pneumoniae*, *K. oxytoca*, *P. aeruginosa*, *P. mirabilis*, *S. boydii*, *S. sonnei*, *E. cloacae*. These nanofibers exhibited a strong biocidal effect on the bacteria and it was suggested that they can be used as efficient antimicrobial materials in contaminated water systems. Figure 6 presents the effect of electrospun TiO_2_ NPs on the morphological aspects of *P. aeruginosa* tested by scanning electron microscopy.

Undoped and Ag-doped TiO_2_ nanofibers were synthesized by an electrospinning process with polyvinylpyrrolidone and Ti tetraisopropoxide as precursors [136]. The effects of the Ag doping ratio (3, 6, and 9%) were determined. It was found that the percentage inhibition of *P. aeruginosa* adhesion increased from 68·8% for undoped TiO_2_ to 90% for 9% Ag-doped TiO_2_ nanofibers. Jatoi [137] described a nanocomposite based on polyurethane nanofibers containing silver/zinc oxide nanoparticles for antimicrobial applications of wound dressings. Both qualitative and quantitative methods were used to determine the antibacterial activity of the composite nanofibers against *E. coli*, *S. aureus*, and *B. subtilis*. It turned out that the sample contained 8% wt. silver/zinc oxide nanoparticles showed a lethal effect against all tested bacteria.

### 6.2. Metallic Oxides

Titanium dioxide (TiO_2_) is an attractive antimicrobial compound due to its photocatalytic nature. It is well known that this metal oxide is chemically stable, non-toxic, inexpensive, and generally recognized as a safe (GRAS) substance. Previously it was shown that TiO_2_ exhibited excellent antibacterial and antifungal properties against a wide range of microorganisms. These properties can be significantly improved by the presence of TiO_2_ in nanometric form. Titanium dioxide nanoparticles are considered to promote the inactivation of microorganisms due to their strong oxidizing power by generating free radicals such as anionic hydroxyl and superoxide radicals [138,139].

There are several reports of the production of TiO_2_-organic hybrids for various biomedical applications [140]. Currently, there is a trend to produce TiO_2_ nanostructures with controlled morphology using the electrospinning technique of polymeric solution with a Ti-containing source (such as titanium isopropoxide) which is followed by thermal degradation of the polymer matrix [140]. It turned out that electrospinning and/or electrospraying techniques are extremely useful in surface coating/specific generation of nanoparticles in situ or the mass of the polymer matrix [141]. For example, Gupta et al. [142] obtained hybrid nanofibers of poly (lactic acid) (PLA/TiO_2_ by electrospinning protocol. Hydrolyzed titanium precursor has been electrosprayed simultaneously on the continuous electrospun PLA nanofibers surface. The adhered amorphous titania has been transformed into TiO_2_ nanoparticles using the hydrothermal treatment at different times. The antimicrobial activity of the produced hybrids has been tested and it has been shown that this material inhibits the growth of popular bacterial pathogens by at least 70%. In another study [136], undoped and Ag-doped TiO_2_ nanofibers were synthesized through electrospinning using polyvinylpyrrolidone and Ti tetraisopropoxide as precursors. It was found that the percentage inhibition adhesion of *Pseudomonas aeruginosa* bacteria increased from 68% for undoped TiO_2_ to 90% for 9% Ag-doped TiO_2_ nanofibers. Regarding electrospinning for the fabrication of polymeric nanofibers, some researchers have demonstrated the multi-functionality of the mats with nano-TiO_2_ and have also proved the antibacterial activity and potential application in tissue engineering by in vitro approaches. In this sense, Lee et al. prepared PVA/nano-TiO_2_ composite fibers with excellent antibacterial activity against *S. aureus* and *K. pneumoniae*, UV protection, and formaldehyde degradability [143]. 

**Figure 7:**
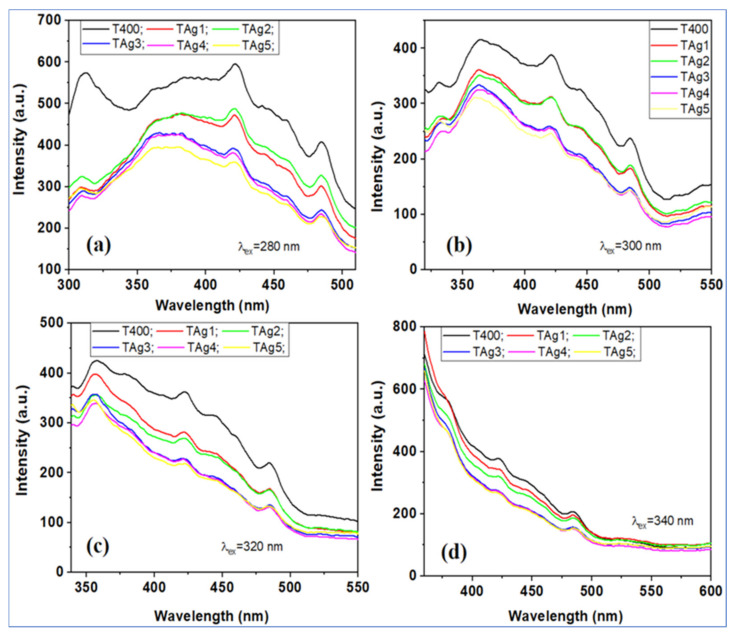
Emission spectra of pure TiO_2_ and Ag–TiO_2_ nanostructured nanofibers at different excitation wavelengths λ_ex_ = 280 nm (**a**), 300 nm (**b**), 320 nm (**c**) and 340 nm (**d**). Reproduced from Ref. [139].

In addition to studying the antimicrobial properties, Pascariu et al. [139] conducted a photoluminescent characterization of the obtained fibers to observe the effect of Ag on the phenomena of recombination and diffusion of carriers in TiO_2_. Figure 7 shows the photoluminescence spectra of the tested materials excited at different wavelengths (*λ_ex_* = 280, 300, 320, and 340 nm). As can be seen, all these fibers present the same emission bands, but with slightly different intensities. In particular, the photoluminescence intensity of nanostructured Ag-TiO_2_ nanofibers was lower compared to pure TiO_2_. The low intensity of the fluorescence spectra suggests that photo-excited electron-hole pairs can be obtained over a longer time, which is beneficial in photocatalytic degradation processes. The presence of Ag nanostructures was thought to have a marked effect on limiting electron-hole recombination, as the photo-excited electron can be captured by Ag nanoparticles, which act as a source of electron storage on the TiO_2_ surface. The presence of silver nanoparticles also contributed significantly to the reduction of the band gap energy and the facilitation of activation by light absorption in the visible region, along with the delay of electron-hole recombination.

Korina et al. [144] studied PHB/TiO_2_ fibrous materials obtained by electrospinning and its combination with electrospraying or impregnation with TiO_2_ nanoparticles. The study of antimicrobial properties revealed that all composite fibers completely killed the bacterium, but those with TiO_2_ on the surface (electrospraying/electrospinning and electrospinning/impregnation) achieved the maximum antibacterial effect in only 30 min. A significant problem in preparing fibrous nanocomposites is preserving their inherent cell adhesion and proliferation. In this aspect, the presence of a relatively high content of TiO_2_ nanoparticles (5–10%) did not interfere with the proliferation of various cell lines [145]. Sundarrajan et al. [146] presented electrospinning of a poly(ethylene imine) (PEI)/nylon blend to obtain polymeric membranes. These membranes were combined with TiO_2_ nanoparticles to degrade chemical and biological warfare (C&B) agents into non-toxic products. The coating of TiO_2_ nanoparticles on the surfaces of nylon and nylon/PEI blend (68/32) nanofibers was performed using the liquid phase deposition (LPD) method. 

Another important inorganic nanomaterial with a strong biocidal effect is zinc oxide (ZnO), which has been used for decades. It should be noted that this metal oxide has a greater ability to kill various types of bacteria than TiO_2_ nanoparticles [147,148]. ZnO-containing electrospun fibers have been described in the literature, but they are mainly limited to polyvinyl alcohol and some natural polymers such as chitosan, cellulose, gelatin, and aliphatic (bio)polyesters such as PLA, PCL, and PHA. For example, PVA/ZnO fibers have been produced by electrospinning a PVA solution containing zinc acetate as a ZnO precursor and evaluated as antibacterial materials, primarily against methicillin-resistant Gram-positive *S. aureus* [148]. However, no antibacterial activity was demonstrated against *P. aeruginosa* and *K. pneumoniae*, which was explained by the differences in the structure of the cell walls of these microorganisms. Lubasowa et al. [149] also designed PVA/ZnO fibers by conventional incorporation of nanoparticles into a polymer solution and used electrospinning to prepare nanofibers. Interestingly, the authors compared the antibacterial activity of ZnO with other metal oxides (TiO_2_, ZrO_2,_ and SnO_2_). The obtained results confirmed that the inclusion of metal oxides imparted antibacterial properties to PVA fibers, but the biocidal properties of PVA mats depended on the type of nanoparticles, and the ability to inhibit bacterial growth was in descending order as follows: ZnO > ZrO_2_ > TiO_2_ > SnO_2_. 

Virovska et al. [150] described the antibacterial properties of PLA fibers with a high content of ZnO nanostructures (23 wt.%). The obtained PLA/ZnO mats showed not only bactericidal properties but also an excellent photocatalytic activity, which was used for the degradation of various pigments (methylene blue and reactive red). PCL is also an important biodegradable polyester whose electrospinning composite mats have been studied by Augustine et al. [151,152]. The authors showed that the diameter of the obtained fibers depended on the content of ZnO nanoparticles (higher content of nanoparticles induced the formation of thicker fibers, which were attributed to changes in conductivity and viscosity). Moreover, it has been shown that a significant antibacterial effect can only be achieved with a ZnO concentration higher than 4 wt.%.

In discussing the antimicrobial properties of metal oxides, it is worth mentioning copper, which is readily available and is one of the most commonly used metals with numerous applications. Moreover, the synthesis and properties of CuO nanostructures have been well documented. However, it seems that there are fewer reports related to the incorporation of CuO nanostructures into polymer fibers by electrospinning, and most of them are only concerned with the influence of nanoparticles on final fiber morphology, whereas the ability to impart antimicrobial properties to electrospun (bio)polymer fibers was hardly considered. The limited use of CuO nanostructures may be attributed to their tendency to oxidation reactions, and thus changes in their general characteristics [153,154]. Haider et al. [153] showed that the presence of a small amount of CuO nanoparticles (0.5% by weight) did not affect the diameter of electrospun fibers based on poly(lactic-co-glycolic acid) (PLGA). The relatively large size of the CuO nanoparticles (diameter 40–100 nm) was sufficient to partially expose them on the polymer fiber surface, which facilitated interactions between inorganic nanoparticles and popular pathogens (*E. coli* and *S. aureus*), and consequently resulting in growth inhibition (>60% for both bacteria). It has been shown that the antimicrobial mechanism was regulated by the release of Cu(II) ions. The authors emphasize that the obtained electrospun mats can be conceived as scaffolds and/or wound dressings with a strong antibacterial effect [153]. Castro-Mayorga et al. [155] developed the PHBHV copolymer films with electrospun PHAs/CuO fibers on their surface, which transmitted unique antibacterial (*Salmonella enterica* and *Listeria monocytogenes*) and antiviral activity (mouse norovirus). Moreover, it was shown that obtained the materials showed good oxygen permeability and mechanical properties. They were completely degraded during composting after 27–30 days, which makes them promising multilayer films for packaging food [155]. In another study, copper nanoparticles were mixed with polyacrylonitrile (PAN) and electrospun into nanofibers (CuPAN nanofibers) [156]. PAN nanofibers containing 1.0, 3.0, and 5.0% copper (*w*/*v*) showed “bead-on-string” morphology with protrusions of copper particles. The diameter of the CuPAN nanofibers varied depending on the copper content, from 386 nm (1.0% *w*/*v* copper) to 922 nm (5.0% *w*/*v* copper). It was proved that filtration of *E. coli* (ETEC) and methicillin-resistant *S. aureus* (MRSA) significantly reduced the number of viable cells of these pathogens. Moreover, the membranes formed with the addition of copper nanoparticles inhibited the growth of enteroaggregative *E. coli* (EAEC), enterohemorrhagic *E. coli* (EHEC), enteroinvasive *E. coli* (EIEC), enteropathogenic *E. coli* (EPEC) as shown with LIVE/DEAD™ BacLight™ staining.

## 7. Electrospun Nanofibers Containing Antimicrobial Plant Extracts

### 7.1. Crude Plant Extracts

In the last 10 years, a lot of research interest has been focused on polymer nanofibers produced by electrospinning with crude plant extracts. These bioactive extracts can be easily obtained by organic solvent extraction from fresh plants or ground dried plants. Crude plant extracts such as Baikalein [157], Centella asiatica [158], green tea [82], Garcinia mangostana [79], Tecomella undulata [159], aloe [160], Grewia mollis [161], chamomile [162], grape seed [163], Indigofera aspalathoides, Azadirachta indica, Memecylon edule and Myristica andamanica [164] have been successfully encapsulated in various electrospun nanofibers. The review by Zhang et al. [165] excellently presents this issue. 

**Figure 8:**
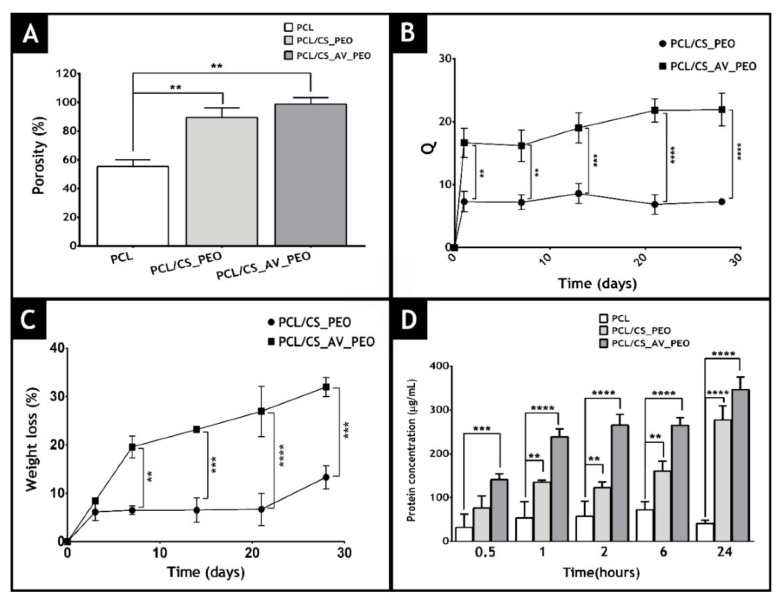
Characterization of the total porosity (**A**), swelling profile (**B**), weight loss (**C**), and protein adsorption (**D**) on the membrane’s surface at different time points. Reproduced from Ref. [166].

Miguel et al. [166] produced an asymmetric membrane that mimics both layers of the skin. It contains an upper dense layer (made of polycaprolactone) that is designed to provide mechanical support to the wound and a lower porous layer (composed of chitosan and Aloe vera) to improve the bactericidal effect of the membrane and ultimately the healing process. The obtained results showed that the produced asymmetric membranes showed porosity, wettability, and mechanical properties similar to those of the native skin. These authors used a liquid displacement method to determine membrane porosity (ethanol was used as displacement fluid). The data obtained are presented in Figure 8 and reveal that the top layer (PCL) displays the lowest porosity (55 ± 5%), which is essential for avoiding microorganism penetration. The bottom layers showed porosities of 89.5 ± 5.3% and 97.8 ± 4.5% for CS/PEO or CS/AV/PEO, respectively. Such results can be explained by the higher number of spaces available between the CS/AV/PEO nanofibers, which have a lower diameter. It was concluded that materials with porosities above 90% are the most appropriate for skin tissue engineering applications since they can provide the required space for cell accommodation, migration, nutrient exchange, and production of a new ECM.

In another study, Baikalein (BAI) has been incorporated into silk fibroin (SFP) and polyvinylpyrrolidone (PVP) fibers and it has been shown that the obtained materials were characterized by antibacterial activity against *S. aureus* (reduction of bacterial viability ranging from 88% to 99%) [157]. Yao et al. [158] produced Centella asiatica (CA) loaded gelatin nanofibers (CA) and showed that the CA extract was effective against *S. aureus*, *E. coli*, and *P. aeruginosa* (these bacteria are commonly involved in wound infections) with MIC of 6.25 mg/mL for *S. aureus* and 25 mg/mL for *E. coli* and *P. aeruginosa*. Sadri et al. [82] studied green tea extract in a chitosan matrix and polyethylene oxide and these authors demonstrated the biocidal activity of the chitosan-PEO/green tea composite fibers against *E. coli* and *S. aureus*, with zones of inhibition on agar plates 4 and 6 mm in diameter, respectively. Suganya et al. [159] have proposed the use of nanofibers of polycaprolactone (PCL) and PVP in which crude bark extracts of Tecomella undulata were embedded for the treatment of skin infections. The potent antibacterial activity of the bark extracts released from the electrospun mats was evaluated against *P. aeruginosa*, *S. aureus*, and *E. coli*, yielding zones of inhibition with diameters of 30, 24, and 28 mm, respectively.

### 7.2. Encapsulation of Essential Oils (EO) into Electrospun Polymeric Fibers

One of the strategies for the fabrication of electrospun materials with antimicrobial properties is the encapsulation of essential oils (EO) into polymeric fibers. These natural chemicals are complex mixtures of volatile compounds that are synthesized by plants for defense and signaling purposes [167]. These compounds protect plants against herbivores and harmful insects and inhibit the growth of pathogenic microorganisms.

Previously, it was shown that various essential oils such as cinnamon [168,169,170], oregano [171,172], mint [173,174], clove [175], thyme [176,177], lavender [33], eucalyptus [178], ginger [179], tea tree [180], manuka [181], black pepper and sage [182] were used to obtain electrospun materials. An excellent review on this subject was provided by Elisa Mele [183].

**Figure 9:**
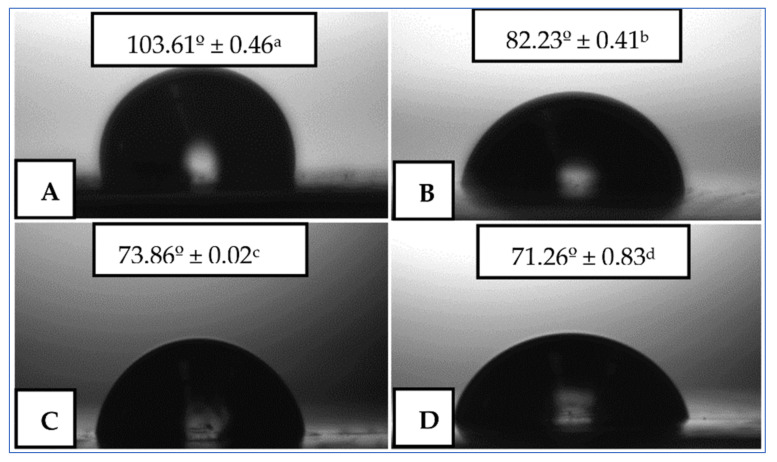
Water contact angle of the electrospun films of: (**A**) Neat poly(3-hydroxybutyrate-co-3-hydroxyvalerate) (PHBV); (**B**) Oregano essential oil (OEO)-containing PHBV; (**C**) Rosemary extract (RE)-containing PHBV; (**D**) Green tea tree extract (GTE)-containing PHBV. Reproduced from Ref. [171].

Some authors point out that the presence of essential oils may affect the surface wettability of the obtained materials [171]. The water contact angle refers to the degree of affinity of water for a surface which determines the degree of hydrophilicity/hydrophobicity of the resultant polymeric material. Figure 9 shows the images of water drops on the membranes, as well as the values of contact angles for electrospun PHBV membranes. As can be seen, the pure PHBV film showed an angle of 103.61°, which is characteristic of hydrophobic materials. The incorporation of the active ingredients resulted in a significant decrease in hydrophobicity. The reduction achieved may be related to the presence of oily particles on the surfaces of the PHBV film, which reduces the surface tension. The hydrophobicity of the material affects the ability of microorganisms to adhere to the surface, and thus affects the efficiency of biofilm formation. It is widely believed that the hydrophobicity of the material surface plays an important role in the initial microbial adhesion and the subsequent biofilm formation [184].

One of the most commonly used essential oil is cinnamon EO, which can be extracted from the leaves and bark of evergreen aromatic trees of the genus Cinnamomum [184]. It is well known that the main chemical component of this oil is cinnamaldehyde, which is active against pathogenic bacteria such as *E. coli*, *S. aureus*, *P. gingivalis*, *L. monocytogenes*, and *B. cereus* [185,186,187,188]. It is believed that cinnamon EO damages the cytoplasmic membrane of both Gram-positive and Gram-negative bacteria. The disruption of membrane integrity results in leakage of nucleic acids and proteins, leading to the death of the bacteria [185,186]. It turned out that Gram-positive bacteria (e.g., *S. aureus*) are more sensitive to the hydrophobic cinnamon oil compared to Gram-negative bacteria. Cinnamon EO has been electrospun in combination with polymers such as polyvinyl alcohol (PVA) [168,169], alginate/PVA [170], polylactic acid (PLA) [189], poly(ethylene oxide) (PEO) [190], and cellulose acetate [191]. It was shown that the received fibers could be used in the food and biomedical sectors. It should be noted that cinnamon EO complexes in combination with cyclodextrins were very often processed. Cyclodextrins are natural cyclic oligosaccharides, characterized by a hydrophilic outer surface and a hydrophobic inner cavity. These structures are widely used to form inclusion complexes with essential oils that are trapped in a hydrophobic cavity to improve the bioavailability and stability of EO.

**Figure 10:**
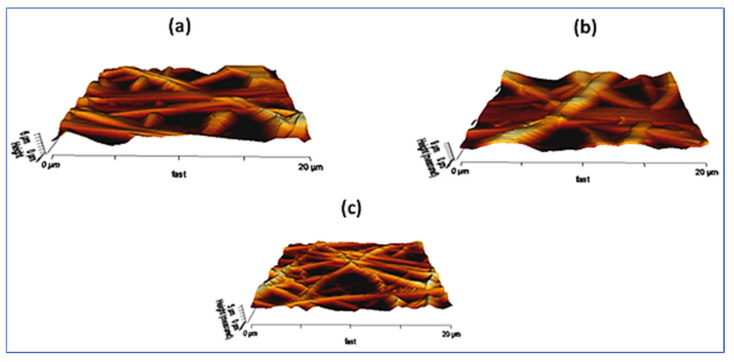
AFM images of (**a**) PU, (**b**) PU/PM, and (**c**) PU/PM/CuSO_4._ Reproduced from Ref. [195].

Recent studies have reported electrospun fibers containing oregano essential oils. The main components of oregano EO are carvacrol and thymol, which have antimicrobial activity against methicillin-resistant *S. aureus* (MRSA), *E. coli*, *B. subtilis*, and *Saccharomyces cerevisiae* [192]. This natural EO, like other essential oils, damages the cell membrane, resulting in the loss of cytosolic material, mainly potassium ions. For example, Ardekani-Zadeh and Hosseini [172] showed that oregano EO can be encapsulated into biodegradable chitosan and poly(caprolactone) (PCL) fibers that were electrospun from formic acid/acetic acid (volume 1:1 ratio) of the solutions [172]. The authors received fibers with a diameter of 210–320 nm containing 1%, 3%, and 5% of oregano EO, which reduced various bacterial populations (*S. aureus*, *L. monocytogenes*, *E. coli*, and *S. enteritidis*) by 40–53% after 3 h of incubation. The essential oil obtained from peppermint (*Mentha piperita*) is rich in menthol/menthon and exhibits antibacterial, antiviral, fungicidal, and anti-inflammatory activity [193]. The effectiveness of EO peppermint against microorganisms was assessed in both liquid and vapor phases, finding that the concentration of monoterpenes (*α*-pinen, *β*-pinen, and limonen) in peppermint EO played the main role in inhibiting bacterial growth by causing extensive cell membrane damage [194]. The electrospun nanofibers enriched with peppermint EO were formed from PCL [173], polyurethane [195], and PEO [196]. Jaganathan et al. [195] analyzed the effect of peppermint oil (PM) and CuSO_4_ incorporation on the surface roughness of electrospun polyurethane (PU) fibers. Representative 3D images of fibrous membranes are shown in Figure 10. The mean roughness (*R_a_*) of flawless PU was 776 ± 468 nm, while PU with the addition of PM and PM/CuSO_4_ has an average roughness of 1039 ± 198 nm and 515 ± 123 nm. Surface roughness measurements showed that the PU/PM fibers are characterized by rougher surfaces, while PU/PM/CuSO4 had smoother surfaces compared to pristine PU. Moreover, electrospun PU/PM fibers showed a larger diameter compared to PU fibers, which may help to improve the surface roughness. On the other hand, electrospun PU/PM/CuSO_4_ showed a better fiber morphology, which resulted in smooth surfaces. The effect of surface roughness on cellular response is still unclear, and the different physicochemical properties of the produced composites may play a role in maintaining pathogenic cell adhesion.

Some authors also described the production of bioactive fibers from polymers that, apart from peppermint EO, contained other bioactive compounds, such as chamomile EO [174]. The electrospun fibers containing only peppermint EO (9% *v*/*v*) showed a high inhibitory effect against *E. coli* and *S. aureus*, whereas the addition of chamomile EO enhanced their antioxidant properties [174]. The main ingredient of clove essential oil is eugenol, which is traditionally used as an analgesic and antiseptic in the prevention and treatment of tooth decay and periodontal disease, acting against cariogenic bacteria such as *S. mutans*, *S. sobrinus*, *P. gingivalis*, and *P. intermedia* [197,198]. The clove EO was encapsulated in electrospun fibers of PCL, gelatin, PCL/gelatin, polyacrylonitrile, alginate/PVA, and polyvinylpyrrolidone and its antimicrobial properties have been studied against *S. aureus*, *E. coli*, *B. subtilis*, *K. pneumoniae*, *C. tropicalis*, and *C. albicans* [183]. For example, PCL/gelatin (7: 3 ratio PCL: gelatin) fibers containing different clove EO concentration (1.5%, 3.0% and 6.0% *v*/*v*) were produced for wound care application [199]. The obtained fibers had an average diameter of 250-300 nm and reduced the viability of *S. aureus* by 30–40% after 6 h of incubation. It turned out that their effectiveness was lower after 24 h of incubation, as the viability of the bacteria returned to a value higher than 90%. A similar phenomenon was observed for *E. coli* exposed to electrospun mats containing 1.5% EO (decrease in cell viability after 6 h, followed by an increase after 24 h). When electrospun fibers containing clove EO at higher concentrations of 3% and 6% *v*/*v* were used in the experiments, a gradual decrease in the viability of *E. coli* to 35–40% was observed after 24 h of exposure. *Thymus vulgaris* L. (commonly known as thyme) has been widely used as an aromatic and medicinal plant in the food, pharmaceutical, and cosmetic industries. It is known that thyme EO has a strong antibacterial and fungicidal effect [200]. The main components of this essential oil are oxidized hydrocarbon monoterpenes and monoterpenes, such as thymol, carvacrol, p-cymene, and γ-terpinene [201]. Until now, several studies of the encapsulation of this oil are known. For example, the encapsulation of EO in poly (vinylpyrrolidone (PVP) and gelatin) fibers has been described by Çallıoğlu et al. [202]. These authors found that PVP/gelatin fiber mats containing 3% *w*/*w* thyme EO were very active against *S. aureus*, *E. coli*, *P. aeruginosa*, and *E. faecalis*. Interestingly, the encapsulated EO retained its antibacterial activity even after 8 days of storage at 24 and 37 °C. Another study [203] showed that the collagen hydrolyzate obtained by the alkaline-enzymatic hydrolysis method and mixed with the thyme EO was used for the fabrication of antimicrobial nanofibers. The obtained fibers showed antimicrobial activity against *S. aureus*, *E. coli*, *P. aeruginosa*, and *C. albicans* [203].

The lavender essential oils are most valued in the pharmaceutical, perfumery, cosmetic, aromatherapy, and phytotherapy industries for their anxiolytic, sedative, anti-inflammatory, antioxidant and antibacterial effects [204]. The main components of these oils are oxidized monoterpenes, such as linalool, linalyl acetate, 1,8-cineole, and camphor [205]. So far, lavender EO has been encapsulated in electrospun fibers of sodium alginate, polyurethane, and polyacrylonitrile to promote wound healing and skin regeneration [183]. For example, polyurethane (Tecoflex) fibers containing various concentrations of silver nanoparticles (1–7% *w*/*w*) and lavender EO (5–20% *w*/*w*) were subjected to electrospinning and these nanocomposite fibers containing 15% and 5% *w*/*w* lavender EO and Ag nanoparticles were effective against *E. coli* and *S. aureus* [33].

## 8. Other Chemical Components

**Figure 11:**
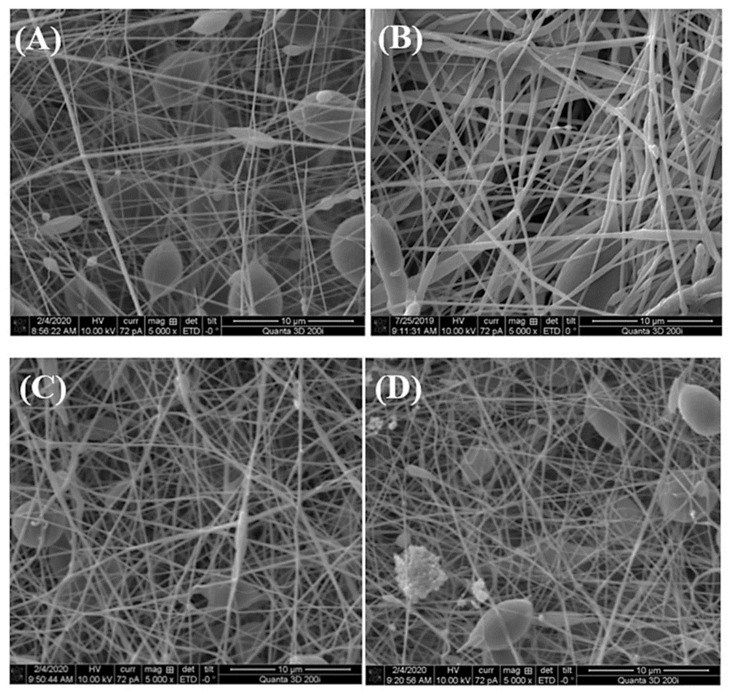
SEM images of electrospun (**A**) pure PCL, (**B**) PCL and 6 wt.% HNT nanofibers, (**C**) PCL and 6 wt.% HNT/ERY (80:20), and (**D**) PCL and 6 wt.% HNT/ERY 60:40. Reproduced from Ref. [206].

Interesting results of the use of halloysite as a nanocarrier for erythromycin (a model antibiotic with a wide range of antibacterial activity) are described by Khunová et al. [206]. PCL and HNT/ERY-based nanofibers were prepared using the electrospinning method (Figure 11), and the antimicrobial activity was assessed as a sterile zone of inhibition around PCL nanofibers containing 7.0 wt.% HNT/ERY. The nanofibers were found to have excellent biocidal activity and inhibited the growth of both Gram-negative (*E. coli*) and Gram-positive (*S. aureus*).

A considerable number of studies are based on the use of pure bioactive compounds isolated from plants such as curcumin and shikonin. Curcumin, a typical hydrophobic polyphenol, is characterized by antioxidant and antimicrobial activity [207]. Recently, studies have been conducted to encapsulate curcumin within electrospun nanofibers by polymers (polycaprolactone, polycaprolactone-polyethylene, polylactide, polyvinyl pyrrolidone) [208,209,210], biopolymers (zein) [211], gelatin [212], and PCL/gelatin [213]. It was reported that obtained materials were characterized by biocidal activity.

Han and co-workers reported on the encapsulation of shikonin into electrospun PCL/poly(trimethylene carbonate) (PTMC) fibers [214]. PCL/PTMC fibers loaded with shikonin were used against *E. coli* and *S. aureus* and it was found that fibers containing 5 wt.% inhibited the growth of these pathogens. Nanofiber-loaded antimicrobial peptides also showed bactericidal activity. Antimicrobial peptides (AMP) are positively charged and can be found in a variety of life forms, including humans and microorganisms [215]. As part of the innate immune response, antimicrobial peptides have broad-spectrum activity against bacterial infections and show a potent therapeutic agent [216]. For example, Song et al. [217] functionalized the surface of silk fibroin nanofibers for the immobilization of AMP (Cys-KR12) from human cathelicidin peptide (LL37). The authors found that Cys-KR12 immobilized on silk fibroin nanofibers inhibited the growth of *S. aureus*, *S. epidermidis*, *E. coli*, and *P. aeruginosa*. Interestingly, the antimicrobial activity of Cys-KR12 was maintained after three weeks [217]. Other examples of antimicrobial electrospun nanofibers are photoactive materials with encapsulated or externally bound photosensitizers and used materials in antimicrobial photodynamic therapy. Suchánek et al. [218] prepared different types of photoactive polymeric nanofiber with porphyrin photosensitizers. These electrospun materials had a stronger antibacterial effect on *E. coli* at a higher temperature. Nylon 6 nanofibers containing organic photosensitizers (benzophenone, 4, 4′-bis(dimethylamino)benzophenone, and thioxanthen-9-one) were also studied to demonstrate the antimicrobial properties in the application of the material to protective clothing and home appliances [219]. 

## 9. Conclusions and Future Trends

Emerging mechanisms of microbial adaptation to conventional antimicrobial agents are a growing problem in the world. Each day there are new understandings and discoveries of new functional materials with antimicrobial properties. Important materials from this point of view are electrospun nanofibers, which are characterized by high surface-to-volume ratio, porous structure, and the ability to be carriers of bioactive compounds. Polymeric nanofibers with antimicrobial properties are attractive in many fields including wound dressing, tissue regeneration, drug delivery, air and water filtration, food protection, and biosensors.

Despite the major findings of antimicrobial electrospun composite fibers, there is currently a need to evaluate their antimicrobial activity against a larger number of microorganisms, since most of the studies have been restricted to two model bacteria, i.e., *Escherichia coli* and *Staphylococcus aureus*. There is only a little information in the available literature data related to fungi, viruses, and other dangerous bacteria.

An important aspect of investigating the potential use of nanofibers for medical and biological purposes is to examine the effect of residual solvents used in the electrospinning process, as often these substances can have toxic properties. Commonly used solvents can be released over a long time, leading to damage to biologically active particles contained in the fiber or harmful effects on living organisms in contact with the nanofiber, e.g., in the fabrication of dressing materials, cell scaffolds, or food storage materials. Meanwhile, one of the most commonly listed advantages of electrospun fibers is their non-toxicity. Unfortunately, studies in this area are often overlooked by many researchers.

Additional studies will be required to provide a deeper understanding of the interactions of these nanomaterials with biofilms as these structures are complex populations that can easily adapt to their surroundings for survival. Moreover, interdisciplinary research involving biology, chemistry, and pharmacology is required for the clinical application of these nanofibers.

## Figures and Tables

**Table 1:** Principal advantages and disadvantages of different methods of fabricating polymeric nanofibers.

Fabrication Method	Advantages	Disadvantages
Drawing	- the simplicity of experimental set-up and minimal equipment requirement - the convenience of the process	- discontinuous process - problem with controlling fiber size
Template synthesis	- simple control of fiber diameter	- the process cannot be scaled
Phase separation	- minimal equipment requirement - nanofiber matrix can be produced	- problem with controlling fiber size - applicable only to particular polymers
Self-assembly	- convenient for producing ultra-fine fibers	- complexity of the process - problem with controlling fiber size - the process cannot be scaled
Electrospinning	- can be fully scaled - cost-effective - excellent repeatability and convenience - continuous and uniform fibers can be produced - simple control of fiber diameter	- instability of the jet

**Table 2:** Application examples for each method of polymer nanofiber fabrication.

Fabrication Method	Structure	Refs.
Drawing	- oriented polyethylene oxide nanofibers with diameters in the range of 200–400 nm - polymer microfiber with high surface smoothness and length uniformity using molten polymethyl methacrylate (PMMA)	[1,6]
Template synthesis	- carbonaceous nanofibers hydrogels and aerogels using Te nanowire template - carbon nanofiber using anodic aluminum oxide template with an average channel diameter of about 25 nm	[7,8]
Phase separation	- nanofibrous scaffolds fabricated from blends of polylactide with functional polymers by a thermally induced phase separation technique	[4]
Self-assembly	- nanofibers from peptide amphiphiles - PPy/TiO_2_/Si composites (TiO_2_ nanorods and polypyrrole (PPy) on the monocrystalline silicon wafer) with hierarchical structures	[3,9]
Electrospinning	- gelatin/nylon (GA/PA66) composite films with diameters from 172.3 to 322.1 nm - polycaprolactone scaffolds coated by the layer-by-layer technology with either - hyaluronic acid or heparin for enhanced regeneration of corneal tissue after surgery	[10,11]

**Table 3:** Antibacterial electrospun fibers (the selected examples).

Electrospun Material	Agent	Microorganism	Refs.
Poly(vinyl alcohol-co-vinyl acetate)/octadecyl amine-montmorillonite	AgNPs	*C. albicans*, *C. tropicalis*, *C. glabrata*, *C. keyfr*, *C. krusei*, *S. aureus*, *E. coli*	[120]
Ethylene vinyl alcohol copolymer	AgNPs	*L. monocytogenes*, *S. enterica*	[121]
Polystyrene (PS)	AgNPs	*S. xylosus*	[122]
Polyvinyl alcohol (PVA)/silk fibroin (SF)	AgNPs	*E. coli*, *S. aureus*	[123]
Ascorbyl palmitate/poly (e-caprolactone) (PCL)	AgNPs	*S. aureus*	[124]
Poly(butylenes succinate)(PBS)	AgNPs	*S. aureus*, *E. coli*	[125]
Polyacrylonitrile	AgNPs	*S. aureus*, *E. coli*, *M. albicans*	[110]
Polycaprolactone	AgNPs	*S. aureus*, *E. coli*, *C. albicans*	[126]
Poly (acrylonitrile-co-methyl methacrylate	AgNPs	*P. aeruginosa*, *S. aureus*, *E.coli*, *Acinetobacter* sp, *K. pneumoniae*, *Micrococcus* sp, *S. epidermidis*, *Candida* sp.	[127]

## Data Availability

Not applicable.

## References

[1] Bu N., Huang Y., Wang X., Yin Z. (2012). Continuously tunable and oriented nanofiber direct-written by mechano-electrospinning. Mater. Manuf. Process..

[2] Tao S.L., Desai T.A. (2007). Aligned arrays of biodegradable poly(ε-caprolactone) nanowires and nanofibers by template synthesis. Nano Lett..

[3] Liao H.S., Lin J., Liu Y., Huang P., Jin A., Chen X. (2016). Self-assembly mechanisms of nanofibers from peptide amphiphiles in solution and on substrate surfaces. Nanoscale.

[4] Li L., Ge J., Wang L., Guo B., Ma P.X. (2014). Electroactive nanofibrous biomimetic scaffolds by thermally induced phase separation. J. Mater. Chem. B.

[5] Zhang C., Li Y., Wang P., Zhang H. (2020). Electrospinning of nanofibers: Potentials and perspectives for active food packaging. Compr. Rev. Food Sci. Food Saf..

[6] Irawati N., Suthaskumar M., John V., Ali N.M., Ahmad H., Harun S.W. (2015). Fabrication of polymer microfiber by direct drawing. Microw. Opt. Technol. Lett..

[7] Liang H.W., Guan Q.F., Chen L.F., Zhu Z., Zhang W.J., Yu S.H. (2012). Macroscopic-scale template synthesis of robust carbonaceous nanofiber hydrogels and aerogels and their applications. Angew. Chemie Int. Ed..

[8] Wang Y., Zheng M., Lu H., Feng S., Ji G., Cao J. (2010). Template Synthesis of Carbon Nanofibers Containing Linear Mesocage Arrays. Nanoscale Res. Lett..

[9] Shi G., Li J., Sang X., Wang L., Ni C., Li Y. (2017). Micro-nano fabrication of hierarchical PPy/TiO 2/Si by continuous self-assembly technology. Mater. Manuf. Process..

[10] Yang Z., Shen C., Zou Y., Wu D., Zhang H., Chen K., Yang Z., Shen C., Zou Y., Wu D. (2021). Application of Solution Blow Spinning for Rapid Fabrication of Gelatin/Nylon 66 Nanofibrous Film. Foods.

[11] Himmler M., Schubert D.W., Dähne L., Egri G., Fuchsluger T.A. (2022). Electrospun PCL Scaffolds as Drug Carrier for Corneal Wound Dressing Using Layer-by-Layer Coating of Hyaluronic Acid and Heparin. Int. J. Mol. Sci..

[12] Miao F., Shao C., Li X., Wang K., Lu N., Liu Y. (2016). Electrospun Carbon Nanofibers/Carbon Nanotubes/Polyaniline Ternary Composites with Enhanced Electrochemical Performance for Flexible Solid-State Supercapacitors. ACS Sustain. Chem. Eng..

[13] Hsu Y.H., Lai C.C., Ho C.L., Lo C.T. (2014). Preparation of interconnected carbon nanofibers as electrodes for supercapacitors. Electrochim. Acta.

[14] Jin J., Shi Z.Q., Wang C.Y. (2014). Electrochemical Performance of Electrospun carbon nanofibers as free-standing and binder-free anodes for Sodium-Ion and Lithium-Ion Batteries. Electrochim. Acta.

[15] Bognitzki M., Hou H., Ishaque M., Frese T., Hellwig M., Schwarte C., Schaper A., Wendorff J.H., Greiner A. (2000). Polymer, metal, and hybrid nano- and mesotubes by coating degradable polymer template fibers (TUFT process). Adv. Mater..

[16] Liu W., Graham M., Evans E.A., Reneker D.H. (2002). Poly(meta-phenylene isophthalamide) nanofibers: Coating and post processing. J. Mater. Res..

[17] Hou H., Jun Z., Reuning A., Schaper A., Wendorff J.H., Greiner A. (2002). Poly(p-xylylene) Nanotubes by Coating and Removal of Ultrathin Polymer Template Fibers. Macromolecules.

[18] Song J., Deng Q., Huang M., Kong Z. (2022). Carbon nanotube enhanced membrane distillation for salty and dyeing wastewater treatment by electrospinning technology. Environ. Res..

[19] Xu D., Chen Y., Qiu T., Qi S., Zhang L., Yin M., Ge K., Wei X., Tian X., Wang P. (2021). Hierarchical mesoporous SnO_2_ nanotube templated by staphylococcus aureus through electrospinning for highly sensitive detection of triethylamine. Mater. Sci. Semicond. Process..

[20] Ahmad A., Khan M.A., Nazir A., Arshad S.N., Qadir M.B., Khaliq Z., Khan Z.S., Satti A.N., Mushtaq B., Shahzad A. (2021). Triaxial electrospun mixed-phased TiO_2_ nanofiber-in-nanotube structure with enhanced photocatalytic activity. Microporous Mesoporous Mater..

[21] Chang C., Tran V.H., Wang J., Fuh Y.-K., Lin L. (2010). Direct-Write Piezoelectric Polymeric Nanogenerator with High Energy Conversion Efficiency. Nano Lett..

[22] Mansouri S., Sheikholeslami T.F., Behzadmehr A. (2019). Investigation on the electrospun PVDF/NP-ZnO nanofibers for application in environmental energy harvesting. J. Mater. Res. Technol..

[23] MacDiarmid A.G., Jones W.E., Norris I.D., Gao J., Johnson A.T., Pinto N.J., Hone J., Han B., Ko F.K., Okuzaki H. (2001). Electrostatically-generated nanofibers of electronic polymers. Synth. Met..

[24] Gandavadi D., Sundarrajan S., Ramakrishna S. (2019). Bio-Based Nanofibers Involved in Wastewater Treatment. Macromol. Mater. Eng..

[25] Wassel A.R., El-Naggar M.E., Shoueir K. (2020). Recent advances in polymer/metal/metal oxide hybrid nanostructures for catalytic applications: A review. J. Environ. Chem. Eng..

[26] Sharma D., Satapathy B.K. (2020). Optimization and physical performance evaluation of electrospun nanofibrous mats of PLA, PCL and their blends. J. Ind. Text..

[27] Kim B.J., Cheong H., Choi E.S., Yun S.H., Choi B.H., Park K.S., Kim I.S., Park D.H., Cha H.J. (2017). Accelerated skin wound healing using electrospun nanofibrous mats blended with mussel adhesive protein and polycaprolactone. J. Biomed. Mater. Res. Part A.

[28] De Carvalho L.D., Peres B.U., Maezono H., Shen Y., Haapasalo M., Jackson J., Carvalho R.M., Manso A.P. (2019). Doxycycline release and antibacterial activity from PMMA/PEO electrospun fiber mats. J. Appl. Oral Sci..

[29] Kurpinski K., Patel S. (2011). Dura mater regeneration with a novel synthetic, bilayered nanofibrous dural substitute: An experimental study. Nanomedicine.

[30] Shoueir K., Kandil S., El-hosainy H., El-Kemary M. (2019). Tailoring the surface reactivity of plasmonic Au@TiO_2_ photocatalyst bio-based chitosan fiber towards cleaner of harmful water pollutants under visible-light irradiation. J. Clean. Prod..

[31] Al-Ahmed Z.A., Al Jahdaly B.A., Radwan H.A., Hassana A.A., Almahri A., Ahmed M.K., Taher M.M. (2021). Electrospun nanofibrous scaffolds of ϵ-polycaprolactone containing graphene oxide and encapsulated with magnetite nanoparticles for wound healing utilizations. Mater. Res. Express.

[32] Farboudi A., Mahboobnia K., Chogan F., Karimi M., Askari A., Banihashem S., Davaran S., Irani M. (2020). UiO-66 metal organic framework nanoparticles loaded carboxymethyl chitosan/poly ethylene oxide/polyurethane core-shell nanofibers for controlled release of doxorubicin and folic acid. Int. J. Biol. Macromol..

[33] Sofi H.S., Akram T., Tamboli A.H., Majeed A., Shabir N., Sheikh F.A. (2019). Novel lavender oil and silver nanoparticles simultaneously loaded onto polyurethane nanofibers for wound-healing applications. Int. J. Pharm..

[34] Ullah A., Ullah S., Khan M.Q., Hashmi M., Nam P.D., Kato Y., Tamada Y., Kim I.S. (2020). Manuka honey incorporated cellulose acetate nanofibrous mats: Fabrication and in vitro evaluation as a potential wound dressing. Int. J. Biol. Macromol..

[35] Lee C.H., Liu K.S., Cheng C.W., Chan E.C., Hung K.C., Hsieh M.J., Chang S.H., Fu X., Juang J.H., Hsieh I.C. (2020). Codelivery of Sustainable Antimicrobial Agents and Platelet-Derived Growth Factor via Biodegradable Nanofibers for Repair of Diabetic Infectious Wounds. ACS Infect. Dis..

[36] Kalantari K., Afifi A.M., Jahangirian H., Webster T.J. (2019). Biomedical applications of chitosan electrospun nanofibers as a green polymer—Review. Carbohydr. Polym..

[37] Cordoba A., Saldias C., Urz M., Montalti M., Guernelli M., Focarete M.L., Leiva A. (2022). On the Versatile Role of Electrospun Polymer Nanofibers as Photocatalytic Hybrid Materials Applied to Contaminated Water Remediation: A Brief Review. Nanomaterials.

[38] Greiner A., Wendorff J.H. (2007). Electrospinning: A fascinating method for the preparation of ultrathin fibers. Angew. Chemie-Int. Ed..

[39] Cheng T., Hund R.D., Aibibu D., Horakova J., Cherif C. (2013). Pure chitosan and chitsoan/chitosan lactate blended nanofibres made by single step electrospinning. Autex Res. J..

[40] Steyaert I., Van Der Schueren L., Rahier H., De Clerck K. (2012). An alternative solvent system for blend electrospinning of polycaprolactone/chitosan nanofibres. Macromol. Symp..

[41] Xu J., Zhang J., Gao W., Liang H., Wang H., Li J. (2009). Preparation of chitosan/PLA blend micro/nanofibers by electrospinning. Mater. Lett..

[42] Pakravan M., Heuzey M.C., Ajji A. (2011). A fundamental study of chitosan/PEO electrospinning. Polymer.

[43] Chen Z., Mo X., Qing F. (2007). Electrospinning of collagen-chitosan complex. Mater. Lett..

[44] Homayoni H., Ravandi S.A.H., Valizadeh M. (2009). Electrospinning of chitosan nanofibers: Processing optimization. Carbohydr. Polym..

[45] Peesan M., Rujiravanit R., Supaphol P. (2006). Electrospinning of hexanoyl chitosan/polylactide blends. J. Biomater. Sci. Polym. Ed..

[46] Kriegel C., Arrechi A., Kit K., Mcclements D.J., Weiss J. (2008). Fabrication, Functionalization, and Application of Electrospun Biopolymer Nanofibers. Crit. Rev. Food Sci. Nutr..

[47] Sharma D., Saha S., Satapathy B.K. (2022). Recent advances in polymer scaffolds for biomedical applications. J. Biomater. Sci. Polym. Ed..

[48] Huang Z.M., Zhang Y.Z., Kotaki M., Ramakrishna S. (2003). A review on polymer nanofibers by electrospinning and their applications in nanocomposites. Compos. Sci. Technol..

[49] Doshi J., Reneker D.H. (1995). Electrospinning process and applications of electrospun fibers. J. Electrostat..

[50] Bhardwaj N., Kundu S.C. (2010). Electrospinning: A fascinating fiber fabrication technique. Biotechnol. Adv..

[51] Ding B., Yu J. (2014). Electrospun Nanofibers for Energy and Environmental Applications.

[52] Balgis R., Kartikowati C.W., Ogi T., Gradon L., Bao L., Seki K., Okuyama K. (2015). Synthesis and evaluation of straight and bead-free nanofibers for improved aerosol filtration. Chem. Eng. Sci..

[53] Megelski S., Stephens J.S., Bruce Chase D., Rabolt J.F. (2002). Micro- and nanostructured surface morphology on electrospun polymer fibers. Macromolecules.

[54] Chan Z., Chen Z., Zhang A., Hu J., Wang X., Yang Z. (2016). Electrospun nanofibers for cancer diagnosis and therapy. Biomater. Sci..

[55] Nam J., Huang Y., Agarwal S., Lannutti J. (2008). Materials selection and residual solvent retention in biodegradable electrospun fibers. J. Appl. Polym. Sci..

[56] D’Amato A.R., Schaub N.J., Cardenas J.M., Franz E., Rende D., Ziemba A.M., Gilbert R.J. (2017). Evaluation of procedures to quantify solvent retention in electrospun fibers and facilitate solvent removal. Fibers Polym..

[57] D’Amato A.R., Schaub N.J., Cardenas J.M., Fiumara A.S., Troiano P.M., Fischetti A., Gilbert R.J. (2017). Removal of retained electrospinning solvent prolongs drug release from electrospun PLLA fibers. Polymer.

[58] Puertas-Bartolomé M., Mora-Boza A., García-Fernández L. (2021). Emerging Biofabrication Techniques: A Review on Natural Polymers for Biomedical Applications. Polymers.

[59] Luraghi A., Peri F., Moroni L. (2021). Electrospinning for drug delivery applications: A review. J. Control. Release.

[60] Hamdan N., Yamin A., Hamid S.A., Khodir W.K.W.A., Guarino V. (2021). Functionalized Antimicrobial Nanofibers: Design Criteria and Recent Advances. J. Funct. Biomater..

[61] Sorlier P., Denuzière A., Viton C., Domard A. (2001). Relation between the degree of acetylation and the electrostatic properties of chitin and chitosan. Biomacromolecules.

[62] Rabea E.I., Badawy M.E.T., Stevens C.V., Smagghe G., Steurbaut W. (2003). Chitosan as antimicrobial agent: Applications and mode of action. Biomacromolecules.

[63] Kong M., Chen X.G., Xing K., Park H.J. (2010). Antimicrobial properties of chitosan and mode of action: A state of the art review. Int. J. Food Microbiol..

[64] Confederat L.G., Tuchilus C.G., Dragan M., Sha’at M., Dragostin O.M. (2021). Preparation and Antimicrobial Activity of Chitosan and Its Derivatives: A Concise Review. Molecules.

[65] Neamnark A., Rujiravanit R., Supaphol P. (2006). Electrospinning of hexanoyl chitosan. Carbohydr. Polym..

[66] Antaby E., Klinkhammer K., Sabantina L. (2021). Electrospinning of chitosan for antibacterial applications—Current trends. Appl. Sci..

[67] Burger C., Hsiao B.S., Chu B. (2006). Nanofibrous materials and their applications. Annu. Rev. Mater. Res..

[68] Cui C., Sun S., Wu S., Chen S., Ma J., Zhou F. (2021). Electrospun chitosan nanofibers for wound healing application. Eng. Regen..

[69] Keirouz A., Radacsi N., Ren Q., Dommann A., Beldi G., Maniura-Weber K., Rossi R.M., Fortunato G. (2020). Nylon-6/chitosan core/shell antimicrobial nanofibers for the prevention of mesh-associated surgical site infection. J. Nanobiotechnology.

[70] Barzegar S., Zare M.R., Shojaei F., Zareshahrabadi Z., Koohi-Hosseinabadi O., Saharkhiz M.J., Iraji A., Zomorodian K., Khorram M. (2021). Core-shell chitosan/PVA-based nanofibrous scaffolds loaded with Satureja mutica or Oliveria decumbens essential oils as enhanced antimicrobial wound dressing. Int. J. Pharm..

[71] Li B., Shan C.L., Zhou Q., Fang Y., Wang Y.L., Xu F., Han L.R., Ibrahim M., Guo L.B., Xie G.L. (2013). Synthesis, characterization, and antibacterial activity of cross-linked chitosan-glutaraldehyde. Mar. Drugs.

[72] Grkovic M., Stojanovic D.B., Pavlovic V.B., Rajilic-Stojanovic M., Bjelovic M., Uskokovic P.S. (2017). Improvement of mechanical properties and antibacterial activity of crosslinked electrospun chitosan/poly (ethylene oxide) nanofibers. Compos. Part B Eng..

[73] Naz M., Jabeen S., Gull N., Ghaffar A., Islam A., Rizwan M., Abdullah H., Rasool A., Khan S., Khan R. (2022). Novel Silane Crosslinked Chitosan Based Electrospun Nanofiber for Controlled Release of Benzocaine. Front. Mater..

[74] Zhou Y., Yang D., Chen X., Xu Q., Lu F., Nie J. (2008). Electrospun water-soluble carboxyethyl chitosan/poly(vinyl alcohol) nanofibrous membrane as potential wound dressing for skin regeneration. Biomacromolecules.

[75] Abdelgawad A.M., Hudson S.M., Rojas O.J. (2014). Antimicrobial wound dressing nanofiber mats from multicomponent (chitosan/silver-NPs/polyvinyl alcohol) systems. Carbohydr. Polym..

[76] Abrigo M., McArthur S.L., Kingshott P. (2014). Electrospun nanofibers as dressings for chronic wound care: Advances, challenges, and future prospects. Macromol. Biosci..

[77] Woo C.H., Choi Y.C., Choi J.S., Lee H.Y., Cho Y.W. (2015). A bilayer composite composed of TiO_2_-incorporated electrospun chitosan membrane and human extracellular matrix sheet as a wound dressing. J. Biomater. Sci. Polym. Ed..

[78] Wang Y., Zhang Q., Zhang C.L., Li P. (2012). Characterisation and cooperative antimicrobial properties of chitosan/nano-ZnO composite nanofibrous membranes. Food Chem..

[79] Suwantong O., Pankongadisak P., Deachathai S., Supaphol P. (2014). Electrospun poly(l-lactic acid) fiber mats containing crude Garcinia mangostana extracts for use as wound dressings. Polym. Bull..

[80] Zupančič Š., Potrč T., Baumgartner S., Kocbek P., Kristl J. (2016). Formulation and evaluation of chitosan/polyethylene oxide nanofibers loaded with metronidazole for local infections. Eur. J. Pharm. Sci..

[81] Sadri M., Sorkhi S.A. (2017). Preparation and characterization of CS/PEO/cefazolin nanofibers with in vitro and in vivo testing. Nanomedicine Res. J..

[82] Sadri M., Arab-Sorkhi S., Vatani H., Bagheri-Pebdeni A. (2015). New wound dressing polymeric nanofiber containing green tea extract prepared by electrospinning method. Fibers Polym..

[83] Lin L., Gu Y., Cui H. (2018). Novel electrospun gelatin-glycerin-ε-Poly-lysine nanofibers for controlling Listeria monocytogenes on beef. Food Packag. Shelf Life.

[84] Kikionis S., Ioannou E., Konstantopoulou M., Roussis V. (2017). Electrospun Micro/Nanofibers as Controlled Release Systems for Pheromones of Bactrocera oleae and Prays oleae. J. Chem. Ecol..

[85] Wang K., Ma Q., Wang S.D., Liu H., Zhang S.Z., Bao W., Zhang K.Q., Ling L.Z. (2016). Electrospinning of silver nanoparticles loaded highly porous cellulose acetate nanofibrous membrane for treatment of dye wastewater. Appl. Phys. A Mater. Sci. Process..

[86] Kalwar K., Shen M. (2019). Electrospun cellulose acetate nanofibers and Au@AgNPs for antimicrobial activity-A mini review. Nanotechnol. Rev..

[87] Shi D., WANG F., Lan T., Zhang Y., SHAO Z. (2016). Convenient fabrication of carboxymethyl cellulose electrospun nanofibers functionalized with silver nanoparticles. Cellulose.

[88] Kalwar K., Hu L., Li D.L., Shan D. (2018). AgNPs incorporated on deacetylated electrospun cellulose nanofibers and their effect on the antimicrobial activity. Polym. Adv. Technol..

[89] Czapka T., Winkler A., Maliszewska I., Kacprzyk R. (2021). Fabrication of photoactive electrospun cellulose acetate nanofibers for antibacterial applications. Energies.

[90] Abdul Khodir W., Abdul Razak A., Ng M., Guarino V., Susanti D. (2018). Encapsulation and Characterization of Gentamicin Sulfate in the Collagen Added Electrospun Nanofibers for Skin Regeneration. J. Funct. Biomater..

[91] Thenmozhi S., Dharmaraj N., Kadirvelu K., Kim H.Y. (2017). Electrospun nanofibers: New generation materials for advanced applications. Mater. Sci. Eng. B Solid-State Mater. Adv. Technol..

[92] Kim K., Luu Y.K., Chang C., Fang D., Hsiao B.S., Chu B., Hadjiargyrou M. (2004). Incorporation and controlled release of a hydrophilic antibiotic using poly(lactide-co-glycolide)-based electrospun nanofibrous scaffolds. J. Control. Release.

[93] Bhattacharjee S. (2021). Understanding the burst release phenomenon: Toward designing effective nanoparticulate drug-delivery systems. Ther. Deliv..

[94] Del Valle L.J., Franco L., Katsarava R., Puiggalí J. (2016). Electrospun biodegradable polymers loaded with bactericide agents. AIMS Mol. Sci..

[95] Chen L., Bromberg L., Hatton T.A., Rutledge G.C. (2008). Electrospun cellulose acetate fibers containing chlorhexidine as a bactericide. Polymer.

[96] Stevenson C.L., Santini J.T., Langer R. (2012). Reservoir-based drug delivery systems utilizing microtechnology. Adv. Drug Deliv. Rev..

[97] Zhang C.L., Yu S.H. (2014). Nanoparticles meet electrospinning: Recent advances and future prospects. Chem. Soc. Rev..

[98] Mahanta N., Valiyaveettil S. (2011). Surface modified electrospun poly(vinyl alcohol) membranes for extracting nanoparticles from water. Nanoscale.

[99] Chen R., Zhao S., Han G., Dong J. (2008). Fabrication of the silver/polypyrrole/polyacrylonitrile composite nanofibrous mats. Mater. Lett..

[100] Shi W., Song S., Zhang H. (2013). Hydrothermal synthetic strategies of inorganic semiconducting nanostructures. Chem. Soc. Rev..

[101] Wu H., Kong D., Ruan Z., Hsu P.C., Wang S., Yu Z., Carney T.J., Hu L., Fan S., Cui Y. (2013). A transparent electrode based on a metal nanotrough network. Nat. Nanotechnol..

[102] Prasher P., Singh M., Mudila H. (2018). ·Silver nanoparticles as antimicrobial therapeutics: Current perspectives and future challenges. 3 Biotech.

[103] Rai M.K., Deshmukh S.D., Ingle A.P., Gade A.K. (2012). Silver nanoparticles: The powerful nanoweapon against multidrug-resistant bacteria. J. Appl. Microbiol..

[104] Naganthran A., Verasoundarapandian G., Khalid F.E., Masarudin M.J., Zulkharnain A., Nawawi N.M., Karim M., Abdullah C.A.C., Ahmad S.A. (2022). Synthesis, Characterization and Biomedical Application of Silver Nanoparticles. Materials.

[105] Duval R.E., Gouyau J., Lamouroux E. (2019). Limitations of recent studies dealing with the antibacterial properties of silver nanoparticles: Fact and opinion. Nanomaterials.

[106] Son W.K., Youk J.H., Park W.H. (2006). Antimicrobial cellulose acetate nanofibers containing silver nanoparticles. Carbohydr. Polym..

[107] Rujitanaroj P.O., Pimpha N., Supaphol P. (2010). Preparation, characterization, and antibacterial properties of electrospun polyacrylonitrile fibrous membranes containing silver nanoparticles. J. Appl. Polym. Sci..

[108] Phan D.N., Dorjjugder N., Saito Y., Taguchi G., Ullah A., Kharaghani D., Kim I.S. (2020). The synthesis of silver-nanoparticle-anchored electrospun polyacrylonitrile nanofibers and a comparison with as-spun silver/polyacrylonitrile nanocomposite membranes upon antibacterial activity. Polym. Bull..

[109] Jatoi A.W., Kim I.S., Ni Q.Q. (2019). A comparative study on synthesis of AgNPs on cellulose nanofibers by thermal treatment and DMF for antibacterial activities. Mater. Sci. Eng. C.

[110] Shi Y., Li Y., Zhang J., Yu Z., Yang D. (2015). Electrospun polyacrylonitrile nanofibers loaded with silver nanoparticles by silver mirror reaction. Mater. Sci. Eng. C.

[111] Liang S., Zhang G., Min J., Ding J., Jiang X. (2014). Synthesis and antibacterial testing of silver/poly (ether amide) composite nanofibers with ultralow silver content. J. Nanomater..

[112] Du L., Li T., Wu S., Zhu H.F., Zou F.Y. (2019). Electrospun composite nanofibre fabrics containing green reduced Ag nanoparticles as an innovative type of antimicrobial insole. RSC Adv..

[113] Yuan J., Geng J., Xing Z., Shen J., Kang I.K., Byun H. (2010). Electrospinning of antibacterial poly(vinylidene fluoride) nanofibers containing silver nanoparticles. J. Appl. Polym. Sci..

[114] Jesús Villarreal-Gómez L., Cornejo-Bravo M., Vera-Graziano R., Grande D. (2016). Electrospinning as a powerful technique for biomedical applications: A critically selected survey. J. Biomater. Sci..

[115] Wang R., Wang Z., Lin S., Deng C., Li F., Chen Z., He H. (2015). Green fabrication of antibacterial polymer/silver nanoparticle nanohybrids by dual-spinneret electrospinning. RSC Adv..

[116] Shi Q., Vitchuli N., Nowak J., Noar J., Caldwell J.M., Breidt F., Bourham M., McCord M., Zhang X. (2011). One-step synthesis of silver nanoparticle-filled nylon 6 nanofibers and their antibacterial properties. J. Mater. Chem..

[117] Tucker N., Stanger J.J., Staiger M.P., Razzaq H., Hofman K. (2012). The History of the Science and Technology of Electrospinning from 1600 to 1995. J. Eng. Fibers Fabr..

[118] Huang S., Zhou L., Li M.C., Wu Q., Kojima Y., Zhou D. (2016). Preparation and properties of electrospun poly (vinyl pyrrolidone)/cellulose nanocrystal/silver nanoparticle composite fibers. Materials.

[119] He M., Chen M., Dou Y., Ding J., Yue H., Yin G., Chen X., Cui Y. (2020). Electrospun silver nanoparticles-embedded feather keratin/poly(vinyl alcohol)/poly(ethylene oxide) antibacterial composite nanofibers. Polymers.

[120] Rzayev Z.M.O., Erdönmez D., Erkan K., Şimşek M., Bunyatova U. (2015). Functional copolymer/Organo-MMT nanoarchitectures. XXII. Fabrication and characterization of antifungal and antibacterial poly (vinyl alcohol-co-vinyl acetate/ODA-MMT/AgNPs nanofibers and nanocoatings by e-spinning and c-spinning methods. Int. J. Polym. Mater. Polym. Biomater..

[121] Martínez-Abad A., Sanchez G., Lagaron J.M., Ocio M.J. (2013). Influence of speciation in the release profiles and antimicrobial performance of electrospun ethylene vinyl alcohol copolymer (EVOH) fibers containing ionic silver ions and silver nanoparticles. Colloid Polym. Sci..

[122] Song J., Wang C., Chen M., Regina V.R., Wang C., Meyer R.L., Xie E., Dong M., Besenbacher F. (2012). Safe and effective Ag nanoparticles immobilized antimicrobial nanononwovens. Adv. Eng. Mater..

[123] Li W., Wang J., Chi H., Wei G., Zhang J., Dai L. (2012). Preparation and antibacterial activity of polyvinyl alcohol/regenerated silk fibroin composite fibers containing Ag nanoparticles. J. Appl. Polym. Sci..

[124] Paneva D., Manolova N., Argirova M., Rashkov I. (2011). Antibacterial electrospun poly(ε-caprolactone)/ascorbyl palmitate nanofibrous materials. Int. J. Pharm..

[125] Tian L., Wang P., Zhao Z., Ji J. (2013). Antimicrobial activity of electrospun poly(butylenes succinate) fiber mats containing PVP-capped silver nanoparticles. Appl. Biochem. Biotechnol..

[126] Dobrzański L.A., Hudecki A., Chladek G., Król W., Mertas A. (2014). Surface properties and antimicrobial activity of composite nanofibers of polycaprolactone with silver precipitations. Arch. Mater. Sci. Eng..

[127] Hafez E.E., El-Aassar M.R., Khalil K.A., Al-Deyab S.S., Taha T.H. (2011). Poly (acrylonitrile-co-methyl methacrylate) nanofibers grafted with bio-nanosilver particles as antimicrobial against multidrug resistant bacteria. African J. Biotechnol..

[128] Gao Y., Truong Y.B., Zhu Y., Louis Kyratzis I. (2014). Electrospun antibacterial nanofibers: Production, activity, and in vivo applications. J. Appl. Polym. Sci..

[129] Lopez-Esparza J., Francisco Espinosa-Cristobal L., Donohue-Cornejo A., Reyes-Lopez S.Y. (2016). Antimicrobial activity of silver nanoparticles in polycaprolactone nanofibers against gram-positive and gram-negative bacteria. Ind. Eng. Chem. Res..

[130] Bhullar S.K., Ruzgar D.G., Fortunato G., Aneja G.K., Orhan M., Saber-Samandari S., Sadighi M., Ahadian S., Ramalingam M. (2019). A Facile Method for Controlled Fabrication of Hybrid Silver Nanoparticle-Poly(-caprolactone) Fibrous Constructs with Antimicrobial Properties. J. Nanosci. Nanotechnol..

[131] Lala N.L., Ramaseshan R., Bojun L., Sundarrajan S., Barhate R.S., Liu Y.J., Ramakrishna S. (2007). Fabrication of nanofibers with antimicrobial functionality used as filters: Protection against bacterial contaminants. Biotechnol. Bioeng..

[132] Castro-Mayorga J.L., Fabra M.J., Cabedo L., Lagaron J.M. (2017). On the use of the electrospinning coating technique to produce antimicrobial polyhydroxyalkanoate materials containing in situ-stabilized silver nanoparticles. Nanomaterials.

[133] Dolina J., Dvořák L., Lederer T., Vacková T., Mikmeková Š., Šlouf M., Černík M. (2016). Characterisation of morphological, antimicrobial and leaching properties of in situ prepared polyurethane nanofibres doped with silver behenate. RSC Adv..

[134] Nthunya L.N., Masheane M.L., Malinga S.P., Barnard T.G., Nxumalo E.N., Mamba B.B., Mhlanga S.D. (2016). UV-assisted reduction of in situ electrospun antibacterial chitosan-based nanofibres for removal of bacteria from water. RSC Adv..

[135] Ansari M.A., Albetran H.M., Alheshibri M.H., Timoumi A., Algarou N.A., Akhtar S., Slimani Y., Almessiere M.A., Alahmari F.S., Baykal A. (2020). Synthesis of electrospun TiO_2_ nanofibers and characterization of their antibacterial and antibiofilm potential against gram-positive and gram-negative bacteria. Antibiotics.

[136] Kudhier M.A., Sabry R.S., Al-Haidarie Y.K., All-Marjani M.F. (2018). Significantly enhanced antibacterial activity of Ag-doped TiO_2_ nanofibers synthesized by electrospinning. Mater. Technol..

[137] Jatoi A.W. (2020). Polyurethane nanofibers incorporated with ZnAg composite nanoparticles for antibacterial wound dressing applications. Compos. Commun..

[138] Azizi-Lalabadi M., Ehsani A., Divband B., Alizadeh-Sani M. (2019). Antimicrobial activity of Titanium dioxide and Zinc oxide nanoparticles supported in 4A zeolite and evaluation the morphological characteristic. Sci. Rep..

[139] Pascariu P., Cojocaru C., Airinei A., Olaru N., Rosca I., Koudoumas E., Suchea M.P. (2021). Innovative ag–tio2 nanofibers with excellent photocatalytic and antibacterial actions. Catalysts.

[140] Shi J., Li J., Wang Y., Zhang C.Y. (2022). TiO_2_-based nanosystem for cancer therapy and antimicrobial treatment: A review. Chem. Eng. J..

[141] Anjusree G.S., Bhupathi A., Balakrishnan A., Vadukumpully S., Subramanian K.R.V., Sivakumar N., Ramakrishna S., Nair S.V., Nair A.S. (2013). Fabricating fiber, rice and leaf-shaped TiO_2_ by tuning the chemistry between TiO_2_ and the polymer during electrospinning. RSC Adv..

[142] Gupta K.K., Mishra P.K., Srivastava P., Gangwar M., Nath G., Maiti P. (2013). Hydrothermal in situ preparation of TiO 2 particles onto poly(lactic acid) electrospun nanofibres. Appl. Surf. Sci..

[143] Lee K., Lee S. (2012). Multifunctionality of poly(vinyl alcohol) nanofiber webs containing titanium dioxide. J. Appl. Polym. Sci..

[144] Korina E., Stoilova O., Manolova N., Rashkov I. (2013). Multifunctional hybrid materials from poly(3-hydroxybutyrate), TiO_2_ nanoparticles, and chitosan oligomers by combining electrospinning/electrospraying and impregnation. Macromol. Biosci..

[145] Braga N.F., Vital D.A., Guerrini L.M., Lemes A.P., Formaggio D.M.D., Tada D.B., Arantes T.M., Cristovan F.H. (2018). PHBV-TiO_2_ mats prepared by electrospinning technique: Physico-chemical properties and cytocompatibility. Biopolymers.

[146] Sundarrajan S., Venkatesan A., Ramakrishna S. (2009). Fabrication of nanostructured self-detoxifying nanofiber membranes that contain active polymeric functional groupsa. Macromol. Rapid Commun..

[147] Espitia P.J.P., de Soares N.F.F., dos Coimbra J.S.R., de Andrade N.J., Cruz R.S., Medeiros E.A.A. (2012). Zinc Oxide Nanoparticles: Synthesis, Antimicrobial Activity and Food Packaging Applications. Food Bioprocess Technol..

[148] Jesline A., John N.P., Narayanan P.M., Vani C., Murugan S. (2015). Antimicrobial activity of zinc and titanium dioxide nanoparticles against biofilm-producing methicillin-resistant Staphylococcus aureus. Appl. Nanosci..

[149] Lubasova D., Barbora S. Antibacterial Efficiency of Nanofiber Membranes with Biologically Active Nanoparticles. Proceedings of the International Conference on Agriculture, Biology and Environmental Sciences.

[150] Virovska D., Paneva D., Manolova N., Rashkov I., Karashanova D. (2014). Electrospinning/electrospraying vs. electrospinning: A comparative study on the design of poly(l-lactide)/zinc oxide non-woven textile. Appl. Surf. Sci..

[151] Augustine R., Dominic E.A., Reju I., Kaimal B., Kalarikkal N., Thomas S. (2014). Electrospun polycaprolactone membranes incorporated with ZnO nanoparticles as skin substitutes with enhanced fibroblast proliferation and wound healing. RSC Adv..

[152] Augustine R., Malik H.N., Singhal D.K., Mukherjee A., Malakar D., Kalarikkal N., Thomas S. (2014). Electrospun polycaprolactone/ZnO nanocomposite membranes as biomaterials with antibacterial and cell adhesion properties. J. Polym. Res..

[153] Haider A., Kwak S., Gupta K.C., Kang I.K. (2015). Antibacterial activity and cytocompatibility of PLGA/CuO hybrid nanofiber scaffolds prepared by electrospinning. J. Nanomater..

[154] Ungur G., Hrůza J. (2015). Influence of copper oxide on the formation of polyurethane nanofibers via electrospinning. Fibers Polym..

[155] Castro Mayorga J.L., Fabra Rovira M.J., Cabedo Mas L., Sánchez Moragas G., Lagarón Cabello J.M. (2018). Antimicrobial nanocomposites and electrospun coatings based on poly(3-hydroxybutyrate-co-3-hydroxyvalerate) and copper oxide nanoparticles for active packaging and coating applications. J. Appl. Polym. Sci..

[156] Ahire J.J., Neveling D.P., Dicks L.M.T. (2018). Polyacrylonitrile (PAN) nanofibres spun with copper nanoparticles: An anti- Escherichia coli membrane for water treatment. Appl. Microbiol. Biotechnol..

[157] Chan W.P., Huang K.C., Bai M.Y. (2017). Silk fibroin protein-based nonwoven mats incorporating baicalein Chinese herbal extract: Preparation, characterizations, and in vivo evaluation. J. Biomed. Mater. Res. Part B Appl. Biomater..

[158] Yao C.H., Yeh J.Y., Chen Y.S., Li M.H., Huang C.H. (2017). Wound-healing effect of electrospun gelatin nanofibres containing Centella asiatica extract in a rat model. J. Tissue Eng. Regen. Med..

[159] Suganya S., Senthil Ram T., Lakshmi B.S., Giridev V.R. (2011). Herbal drug incorporated antibacterial nanofibrous mat fabricated by electrospinning: An excellent matrix for wound dressings. J. Appl. Polym. Sci..

[160] Agnes Mary S., Giri Dev V.R. (2015). Electrospun herbal nanofibrous wound dressings for skin tissue engineering. J. Text. Inst..

[161] Al-Youssef H.M., Amina M., Hassan S., Amna T., Jeong J.W., Nam K.T., Kim H.Y. (2013). Herbal drug loaded poly(D,L-lactide-co-glycolide) ultrafine fibers: Interaction with pathogenic bacteria. Macromol. Res..

[162] Motealleh B., Zahedi P., Rezaeian I., Moghimi M., Abdolghaffari A.H., Zarandi M.A. (2014). Morphology, drug release, antibacterial, cell proliferation, and histology studies of chamomile-loaded wound dressing mats based on electrospun nanofibrous poly(ε-caprolactone)/polystyrene blends. J. Biomed. Mater. Res. Part B Appl. Biomater..

[163] Lin S., Chen M., Jiang H., Fan L., Sun B., Yu F., Yang X., Lou X., He C., Wang H. (2016). Green electrospun grape seed extract-loaded silk fibroin nanofibrous mats with excellent cytocompatibility and antioxidant effect. Colloids Surf. B Biointerfaces.

[164] Jin G., Prabhakaran M.P., Kai D., Annamalai S.K., Arunachalam K.D., Ramakrishna S. (2013). Tissue engineered plant extracts as nanofibrous wound dressing. Biomaterials.

[165] Zhang W., Ronca S., Mele E. (2017). Electrospun nanofibres containing antimicrobial plant extracts. Nanomaterials.

[166] Miguel S.P., Ribeiro M.P., Coutinho P., Correia I.J. (2017). Electrospun polycaprolactone/Aloe Vera_chitosan nanofibrous asymmetric membranes aimed for wound healing applications. Polymers.

[167] Rehman R., Hanif M.A., Mushtaq Z., Al-Sadi A.M. (2016). Biosynthesis of essential oils in aromatic plants: A review. Food Rev. Int..

[168] Wen P., Zhu D.H., Wu H., Zong M.H., Jing Y.R., Han S.Y. (2016). Encapsulation of cinnamon essential oil in electrospun nanofibrous film for active food packaging. Food Control.

[169] Feng K., Wen P., Yang H., Li N., Lou W.Y., Zong M.H., Wu H. (2017). Enhancement of the antimicrobial activity of cinnamon essential oil-loaded electrospun nanofilm by the incorporation of lysozyme. RSC Adv..

[170] Rafiq M., Hussain T., Abid S., Nazir A., Masood R. (2018). Development of sodium alginate/PVA antibacterial nanofibers by the incorporation of essential oils. Mater. Res. Express.

[171] Figueroa-Lopez K.J., Vicente A.A., Reis M.A.M., Torres-Giner S., Lagaron J.M. (2019). Antimicrobial and antioxidant performance of various essential oils and natural extracts and their incorporation into biowaste derived poly(3-hydroxybutyrate-co-3-hydroxyvalerate) layers made from electrospun ultrathin fibers. Nanomaterials.

[172] Hasanpour Ardekani-Zadeh A., Hosseini S.F. (2019). Electrospun essential oil-doped chitosan/poly(ε-caprolactone) hybrid nanofibrous mats for antimicrobial food biopackaging exploits. Carbohydr. Polym..

[173] Unalan I., Slavik B., Buettner A., Goldmann W.H., Frank G., Boccaccini A.R. (2019). Physical and Antibacterial Properties of Peppermint Essential Oil Loaded Poly (ε-caprolactone) (PCL) Electrospun Fiber Mats for Wound Healing. Front. Bioeng. Biotechnol..

[174] Tang Y., Zhou Y., Lan X., Huang D., Luo T., Ji J., Mafang Z., Miao X., Wang H., Wang W. (2019). Electrospun Gelatin Nanofibers Encapsulated with Peppermint and Chamomile Essential Oils as Potential Edible Packaging. J. Agric. Food Chem..

[175] Sahal G., Nasseri B., Ebrahimi A., Bilkay I.S. (2019). Electrospun essential oil-polycaprolactone nanofibers as antibiofilm surfaces against clinical Candida tropicalis isolates. Biotechnol. Lett..

[176] Liu J.X., Dong W.H., Mou X.J., Liu G.S., Huang X.W., Yan X., Zhou C.F., Jiang S., Long Y.Z. (2020). In Situ Electrospun Zein/Thyme Essential Oil-Based Membranes as an Effective Antibacterial Wound Dressing. ACS Appl. Bio Mater..

[177] Lin L., Liao X., Cui H. (2019). Cold plasma treated thyme essential oil/silk fibroin nanofibers against Salmonella Typhimurium in poultry meat. Food Packag. Shelf Life.

[178] Dias Antunes M., da Silva Dannenberg G., Fiorentini Â.M., Pinto V.Z., Lim L.T., da Rosa Zavareze E., Dias A.R.G. (2017). Antimicrobial electrospun ultrafine fibers from zein containing eucalyptus essential oil/cyclodextrin inclusion complex. Int. J. Biol. Macromol..

[179] Da Silva F.T., da Cunha K.F., Fonseca L.M., Antunes M.D., El Halal S.L.M., Fiorentini Â.M., da Zavareze E.R., Dias A.R.G. (2018). Action of ginger essential oil (*Zingiber officinale*) encapsulated in proteins ultrafine fibers on the antimicrobial control in situ. Int. J. Biol. Macromol..

[180] Cui H., Bai M., Lin L. (2018). Plasma-treated poly(ethylene oxide) nanofibers containing tea tree oil/beta-cyclodextrin inclusion complex for antibacterial packaging. Carbohydr. Polym..

[181] Zhang W., Huang C., Kusmartseva O., Thomas N.L., Mele E. (2017). Electrospinning of polylactic acid fibres containing tea tree and manuka oil. React. Funct. Polym..

[182] Wang P., Mele E. (2018). Effect of antibacterial plant extracts on the morphology of electrospun poly(lactic acid) fibres. Materials.

[183] Mele E. (2020). Electrospinning of essential oils. Polymers.

[184] D’agostino M., Tesse N., Frippiat J.P., Machouart M., Debourgogne A. (2019). Essential oils and their natural active compounds presenting antifungal properties. Molecules.

[185] Zhang Y., Liu X., Wang Y., Jiang P., Quek S.Y. (2016). Antibacterial activity and mechanism of cinnamon essential oil against Escherichia coli and Staphylococcus aureus. Food Control.

[186] Huang D.F., Xu J.G., Liu J.X., Zhang H., Hu Q.P. (2014). Chemical constituents, antibacterial activity and mechanism of action of the essential oil from Cinnamomum cassia bark against four food-related bacteria. Microbiology.

[187] Wang Y., Zhang Y., qin Shi Y., hua Pan X., hua Lu Y., Cao P. (2018). Antibacterial effects of cinnamon (Cinnamomum zeylanicum) bark essential oil on Porphyromonas gingivalis. Microb. Pathog..

[188] Goñi P., López P., Sánchez C., Gómez-Lus R., Becerril R., Nerín C. (2009). Antimicrobial activity in the vapour phase of a combination of cinnamon and clove essential oils. Food Chem..

[189] Wen P., Zhu D.H., Feng K., Liu F.J., Lou W.Y., Li N., Zong M.H., Wu H. (2016). Fabrication of electrospun polylactic acid nanofilm incorporating cinnamon essential oil/β-cyclodextrin inclusion complex for antimicrobial packaging. Food Chem..

[190] Lin L., Dai Y., Cui H. (2017). Antibacterial poly(ethylene oxide) electrospun nanofibers containing cinnamon essential oil/beta-cyclodextrin proteoliposomes. Carbohydr. Polym..

[191] Liakos I., Rizzello L., Hajiali H., Brunetti V., Carzino R., Pompa P.P., Athanassiou A., Mele E. (2015). Fibrous wound dressings encapsulating essential oils as natural antimicrobial agents. J. Mater. Chem. B.

[192] Cui H., Zhang C., Li C., Lin L. (2019). Antibacterial mechanism of oregano essential oil. Ind. Crops Prod..

[193] Işcan G., Kirimer N., Kürkcüoǧlu M., Başer K.H.C., Demirci F. (2002). Antimicrobial screening of Mentha piperita essential oils. J. Agric. Food Chem..

[194] Tyagi A.K., Malik A. (2011). Antimicrobial potential and chemical composition of Mentha piperita oil in liquid and vapour phase against food spoiling microorganisms. Food Control.

[195] Jaganathan S.K., Mani M.P., Khudzari A.Z.M. (2019). Electrospun combination of peppermint oil and copper sulphate with conducive physico-chemical properties for wound dressing applications. Polymers.

[196] Suganya Bharathi B., Stalin T. (2019). Cerium oxide and peppermint oil loaded polyethylene oxide/graphene oxide electrospun nanofibrous mats as antibacterial wound dressings. Mater. Today Commun..

[197] Chaieb K., Hajlaoui H., Zmantar T., Ben Kahla-Nakbi A., Rouabhia M., Mahdouani K., Bakhrouf A. (2007). The Chemical Composition and Biological Activity of Clove Essential Oil, Eugenia caryophyllata (Syzigium aromaticum L. Myrtaceae): A Short Review. Phytother. Res.

[198] Moon S.E., Kim H.Y., Cha J.D. (2011). Synergistic effect between clove oil and its major compounds and antibiotics against oral bacteria. Arch. Oral Biol..

[199] Unalan I., Endlein S.J., Slavik B., Buettner A., Goldmann W.H., Detsch R., Boccaccini A.R. (2019). Evaluation of electrospun poly(ε-caprolactone)/gelatin nanofiber mats containing clove essential oil for antibacterial wound dressing. Pharmaceutics.

[200] Rota M.C., Herrera A., Martínez R.M., Sotomayor J.A., Jordán M.J. (2008). Antimicrobial activity and chemical composition of Thymus vulgaris, Thymus zygis and Thymus hyemalis essential oils. Food Control.

[201] Tariq S., Wani S., Rasool W., Shafi K., Bhat M.A., Prabhakar A., Shalla A.H., Rather M.A. (2019). A comprehensive review of the antibacterial, antifungal and antiviral potential of essential oils and their chemical constituents against drug-resistant microbial pathogens. Microb. Pathog..

[202] Çallıoğlu F.C., Güler H.K., Çetin E.S. (2019). Emulsion electrospinning of bicomponent poly (vinyl pyrrolidone)/gelatin nanofibers with thyme essential oil Emulsion electrospinning of bicomponent poly (vinyl pyrrolidone)/gelatin nano fi bers with thyme essential oil. Mater. Res. Express.

[203] Berechet M.D., Gaidau C., Miletic A., Pilic B., Râpă M., Stanca M., Ditu L.-M., Constantinescu R., Lazea-Stoyanova A. (2020). Bioactive Properties of Nanofibres Based on Concentrated Collagen Hydrolysate Loaded with Thyme and Oregano Essential Oils. Materials.

[204] Cavanagh H.M.A., Wilkinson J.M. (2002). Biological activities of lavender essential oil. Phyther. Res..

[205] Aprotosoaie A.C., Gille E., Trifan A., Luca V.S., Miron A. (2017). Essential oils of Lavandula genus: A systematic review of their chemistry. Phytochem. Rev..

[206] Electrospun A., Nanofibers P., Khunová V., Kováčová M., Olejniková P., Ondreáš F., Špitalský Z., Ghosal K., Berkeš D. (2022). Antibacterial Electrospun Polycaprolactone Nanofibers Reinforced by Halloysite Nanotubes for Tissue Engineering. Polymers.

[207] Anand P., Kunnumakkara A.B., Newman R.A., Aggarwal B.B. (2007). Bioavailability of curcumin: Problems and promises. Mol. Pharm..

[208] Yakub G., Toncheva A., Manolova N., Rashkov I., Danchev D., Kussovski V. (2016). Electrospun polylactide-based materials for curcumin release: Photostability, antimicrobial activity, and anticoagulant effect. J. Appl. Polym. Sci..

[209] Sridhar R., Ravanan S., Venugopal J.R., Sundarrajan S., Pliszka D., Sivasubramanian S., Gunasekaran P., Prabhakaran M., Madhaiyan K., Sahayaraj A. (2014). Curcumin-and natural extract-loaded nanofibres for potential treatment of lung and breast cancer: In vitro efficacy evaluation. J. Biomater. Sci. Polym. Ed..

[210] Wang C., Ma C., Wu Z., Liang H., Yan P., Song J., Ma N., Zhao Q. (2015). Enhanced Bioavailability and Anticancer Effect of Curcumin-Loaded Electrospun Nanofiber: In Vitro and In Vivo Study. Nanoscale Res. Lett..

[211] Trung H., Bui H.T., Chung O.H., Park J.S. (2014). Fabrication of Electrospun Antibacterial Curcumin-loaded Zein Nanofibers. Polymer.

[212] Fallah M., Bahrami S.H., Ranjbar-Mohammadi M. (2016). Fabrication and characterization of PCL/gelatin/curcumin nanofibers and their antibacterial properties. J. Ind. Text..

[213] Deng L., Kang X., Liu Y., Feng F., Zhang H. (2017). Effects of surfactants on the formation of gelatin nanofibres for controlled release of curcumin. Food Chem..

[214] Han J., Chen T.X., Branford-White C.J., Zhu L.M. (2009). Electrospun shikonin-loaded PCL/PTMC composite fiber mats with potential biomedical applications. Int. J. Pharm..

[215] Wolfgang M.C., Martin Dozois C., Schmelcher M., Zurich E., Mahlapuu margitmahlapuu M., Mahlapuu M., Håkansson J., Ringstad L., Björn C. (2016). Antimicrobial Peptides: An Emerging Category of Therapeutic Agents. Front. Cell. Infect. Microbiol..

[216] Kang S.J., Park S.J., Mishig-Ochir T., Lee B.J. (2014). Antimicrobial peptides: Therapeutic potentials. Expert Rev. Anti. Infect. Ther..

[217] Song D.W., Kim S.H., Kim H.H., Lee K.H., Ki C.S., Park Y.H. (2016). Multi-biofunction of antimicrobial peptide-immobilized silk fibroin nanofiber membrane: Implications for wound healing. Acta Biomater..

[218] Suchánek J., Henke P., Mosinger J., Zelinger Z., Kubát P. (2014). Effect of temperature on photophysical properties of polymeric nanofiber materials with porphyrin photosensitizers. J. Phys. Chem. B.

[219] Lim K.S., Oh K.W., Kim S.H. (2012). Antimicrobial activity of organic photosensitizers embedded in electrospun nylon 6 nanofibers. Polym. Int..

